# Supplementary material for: The complex underpinnings of genetic background effects
Source: Nat Commun. 2018 Sep 17;9:3548. doi: 10.1038/s41467-018-06023-5 (PMC6141565; doi:10.1038/s41467-018-06023-5)
Supplement: Supplementary file 1 — Supplementary Information [file 41467_2018_6023_MOESM1_ESM.docx]

Supplementary materials for

**The complex underpinnings of genetic background effects**

Martin N. Mullis, Takeshi Matsui, Rachel Schell, Ryan Foree, and Ian M. Ehrenreich

**Supplementary Figures**

**
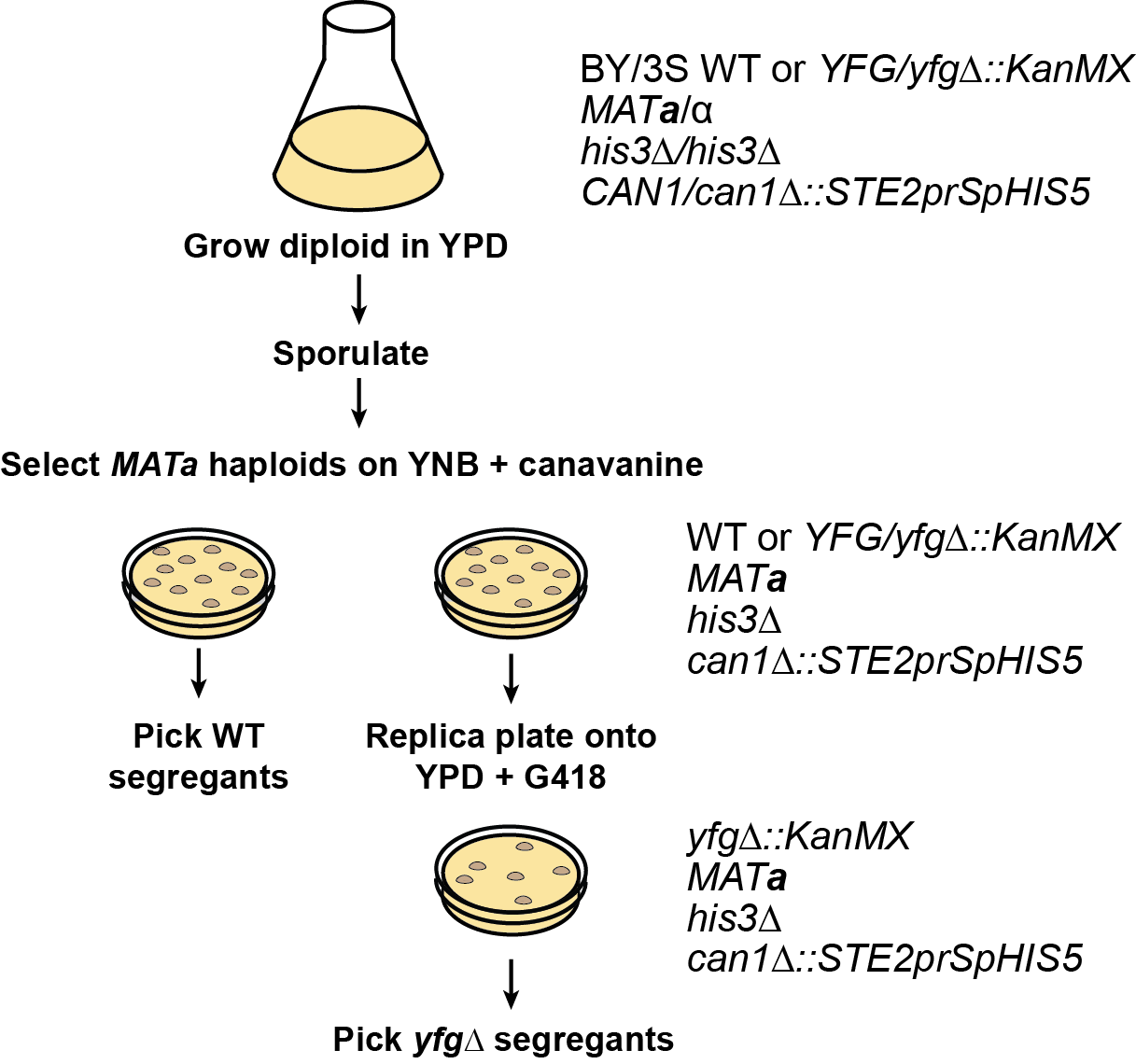
**

**Supplementary Figure 1. Generation of BYx3S knockout segregants.** For each knockout or wild type background in our study, a BY/3S diploid was generated and sporulated. *MAT****a*** segregants were obtained using the synthetic genetic array marker system ^1^ (**Methods**). Wild type segregants were collected directly from *MAT****a*** selection plates, while knockout segregants were replica-plated from *MAT****a*** selection plates onto G418 plates prior to their collection to select for segregants with the gene deletion.


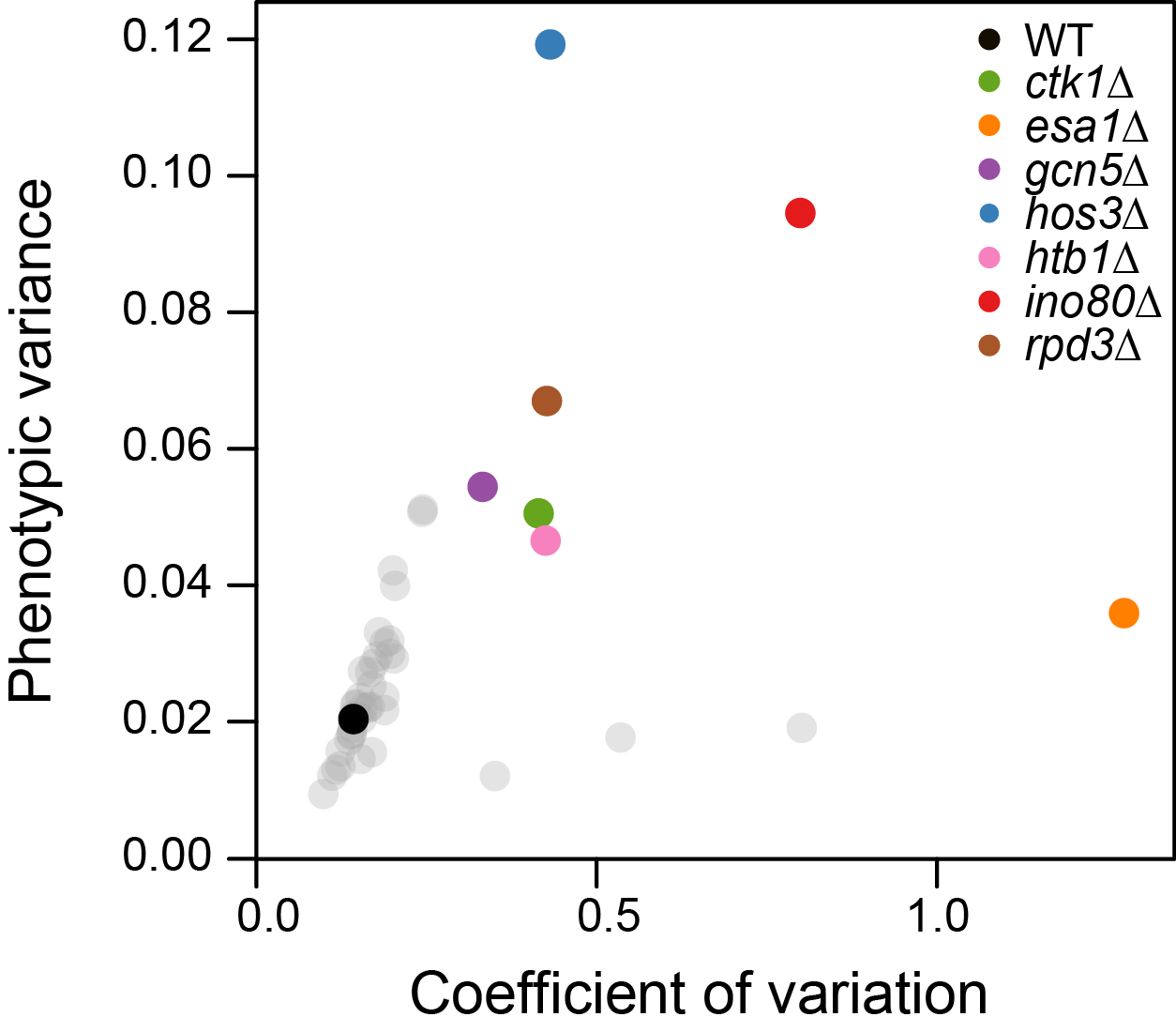


**Supplementary Figure 2.** **Certain genes exhibit significant background effects when perturbed.** In a preliminary screen, we generated and phenotyped segregants from 47 mutant versions of the same yeast cross, each of which lacked a different chromatin-associated protein (**Supplementary Table 1**). The coefficient of variation found in a given knockout background is shown on the x-axis, while the phenotypic variance is shown on the y-axis. In addition to increased phenotypic variance, we found that knockout of certain genes, in particular *ESA1*, caused severe growth reductions in all but few outlier segregants, which resulted in a high coefficient of variation. Presence of these outliers may reflect higher-order interactions among loci, which would lead to small fraction of individuals showing unusual growth ^2,3^. Using Levene’s Test, we found that seven genes exhibit significant background effects when deleted: *CTK1, ESA1, GCN5, HOS3, HTB1, INO80,* and *RPD3* (**Supplementary Table 2**). The points corresponding to these genes are shown in color, while the point corresponding to wild type is illustrated in black. All other points are gray.


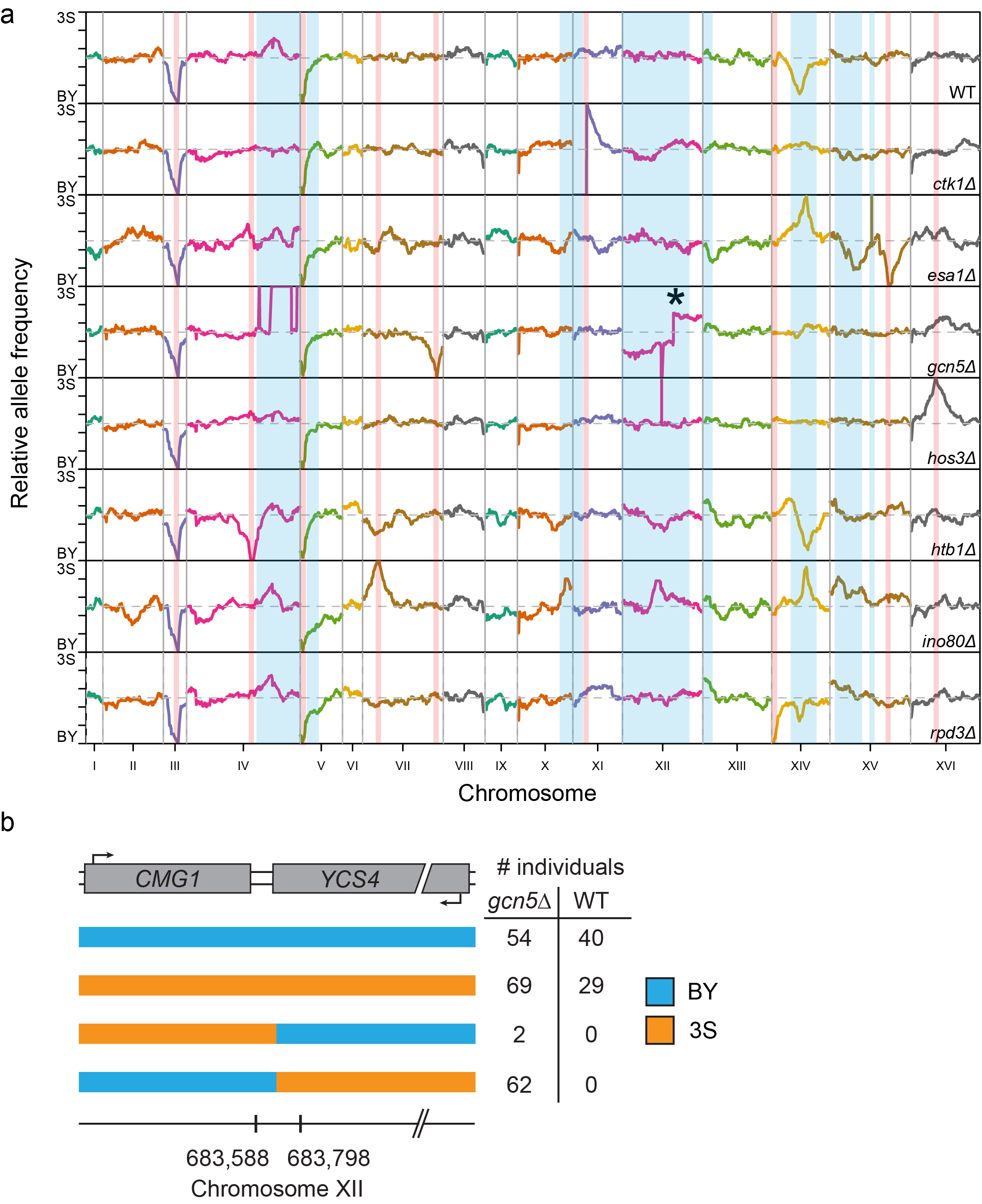


**Supplementary Figure 3. Allele frequency plot.** (**a**) Allele frequency plots are shown for each knockout and wild type background. 50kb regions surrounding the knockouts, as well as regions that were fixed due to selection on markers used to generate *MAT****a*** haploids, are highlighted in red. Regions where the allele frequency in at least one population is significantly different from other populations (**Supplementary Table 4; Supplementary Note 1; Methods**), as well as regions that were fixed due to mitotic recombination or gene conversion in the progenitor hemizygous diploids, are highlighted in blue (**Supplementary Table 5; Supplementary Note 3; Methods**). (**b**) We observed that all of Chromosome XII was enriched in the *gcn5∆* population. This appears to be due to selection against a recombinant version of the chromosome. Specifically, individuals who harbored a 3S-BY recombinant haplotype centered on *YCS4* were depleted (**Supplementary Table 5**). This site of increased recombination on Chromosome XII in the *gcn5*∆ population is denoted with an asterisk. No recombinants were observed at this site among wild type segregants, suggesting that the *gcn5∆* knockout resulted in a new recombination hotspot.

**
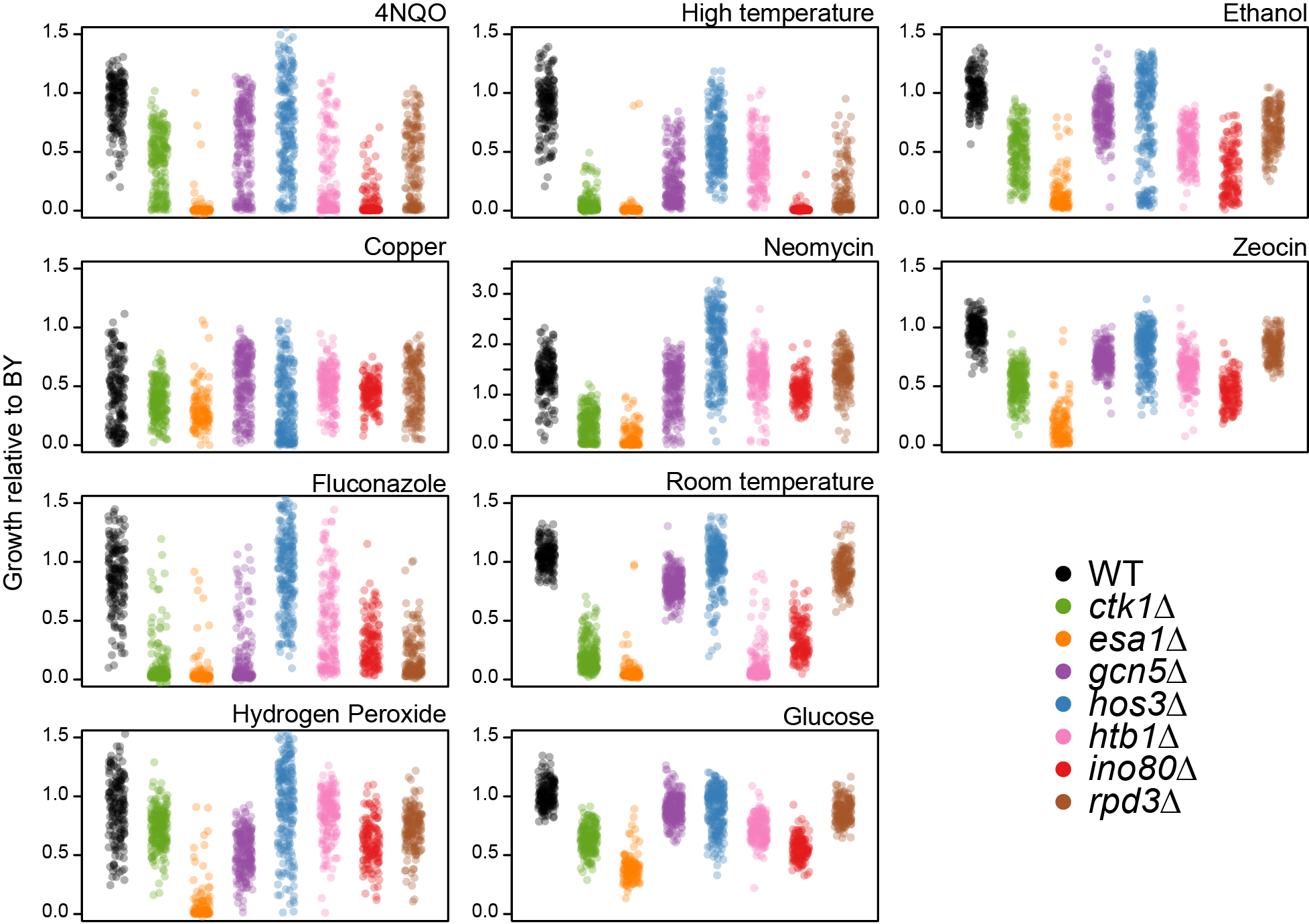
**

**Supplementary Figure 4. Growth of all 1,411 segregants across the 10 environments.**

**
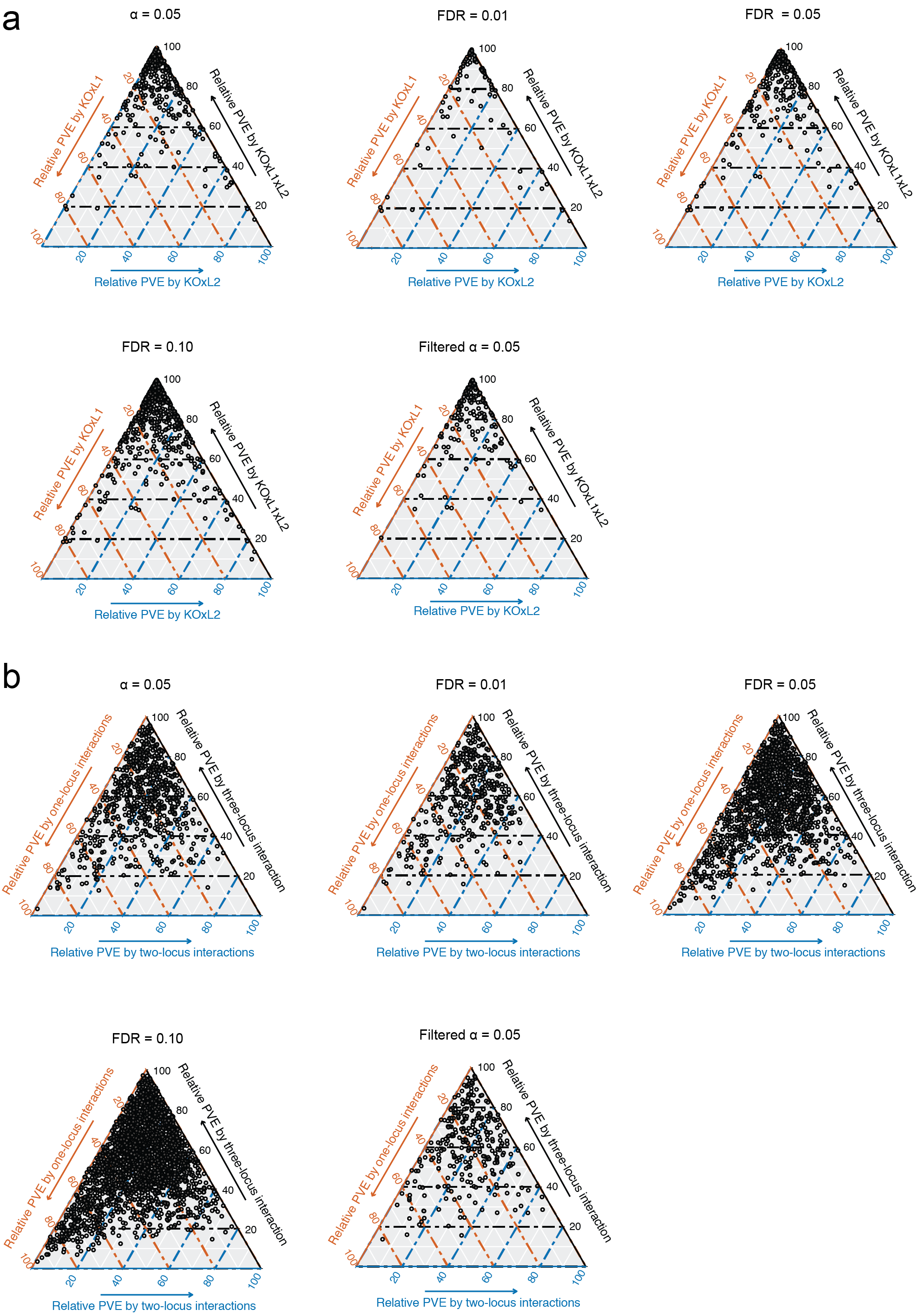
**

**Supplementary Figure 5. Individual and joint contributions of loci to background effects across different significance thresholds.** In **a**, for each mutation-responsive two-locus effect identified across different significance thresholds, the relative phenotypic variance explained (PVE) by the individual involved loci and their interaction is illustrated. The same analysis was also performed after loci that show biased two-locus allele frequencies were filtered from the set of two-locus effects identified at the α = 0.05 threshold. In **b**, for each mutation-responsive three-locus effect identified across different significance thresholds, the relative PVE for the individual loci, the pairs of loci, and the trio of loci is provided. Similar to mutation-responsive two-locus effects, loci that show biased three-locus allele frequencies were filtered from the set of three-locus effects identified at the α = 0.05 threshold and their relative PVE values were determined. Relative PVE values were calculated using sum of squares obtained from ANOVA tables, as described in the **Methods**. As with the results reported in the paper, which were obtained using the α = 0.05 threshold, we find that loci involved in most mutation-responsive effects identified at other threshold mainly contribute to background effects through higher-order epistasis.

**
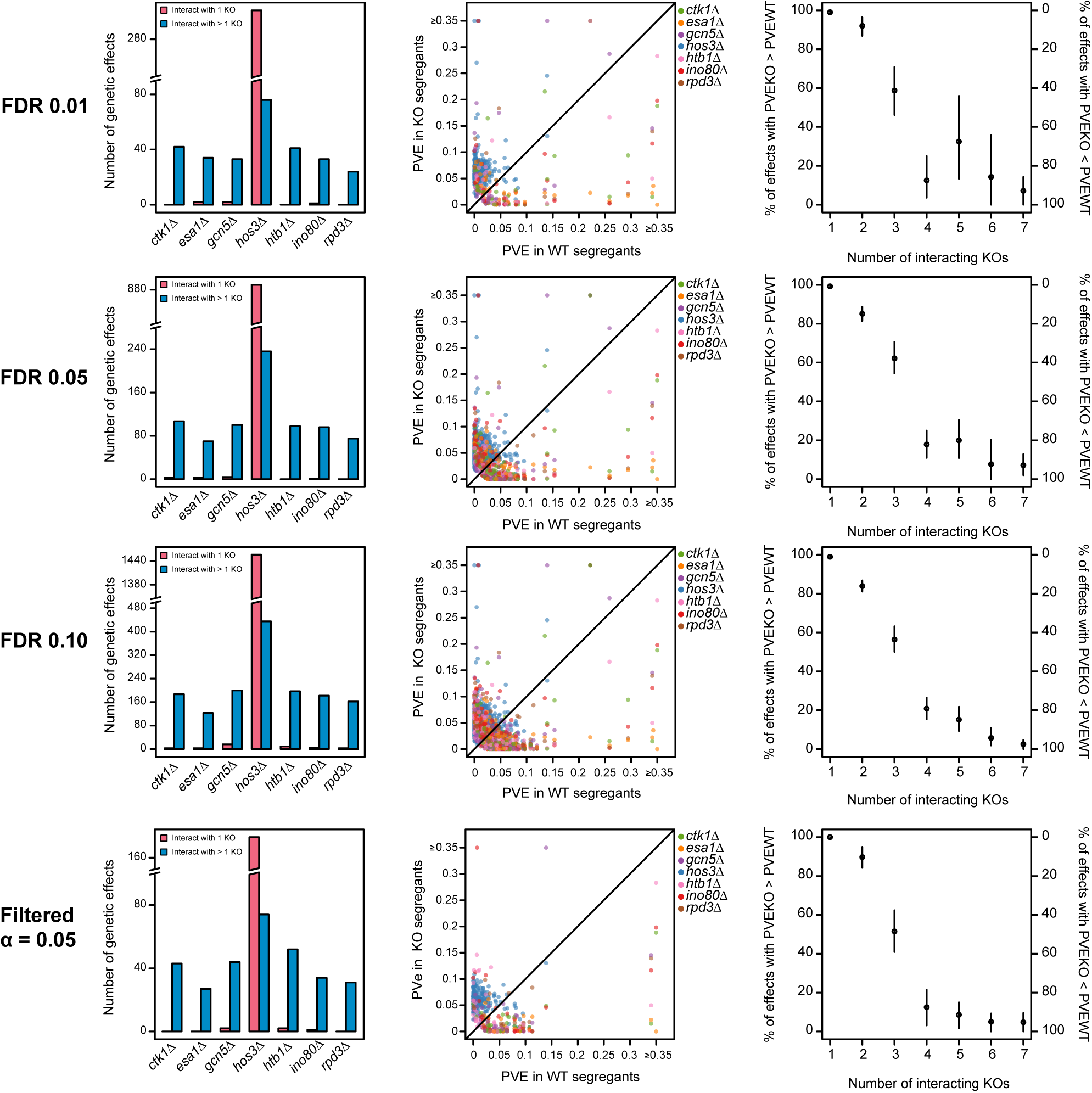
**

**Supplementary Figure 6. Analysis of how mutation-responsive effects interact with different knockouts at multiple significance thresholds. O**ur findings are reported across FDRs of 0.01, 0.05, and 0.10, as well as α = 0.05 after filtering of loci and multi-locus genotype classes with biased frequencies. In each row, the left plot shows the number of genetic effects found in one (pink) or more (blue) knockout backgrounds. The middle plot shows the PVE for mutation-responsive effects in the wild type and relevant knockout segregants. The third plot shows the percentage of genetic effects with larger PVE in the relevant knockout background than the wild type background (PVE_KO_ > PVE_WT_) as a function of the number of knockouts that interact with the effect. We provide an additional y-axis on the right side of the plot, which indicates the percentage of genetic effects with smaller PVE in the relevant knockout background than the wild type background (PVE_KO_  < PVE_WT_) . Error bars represent 95% bootstrap confidence intervals (**Methods**).

**
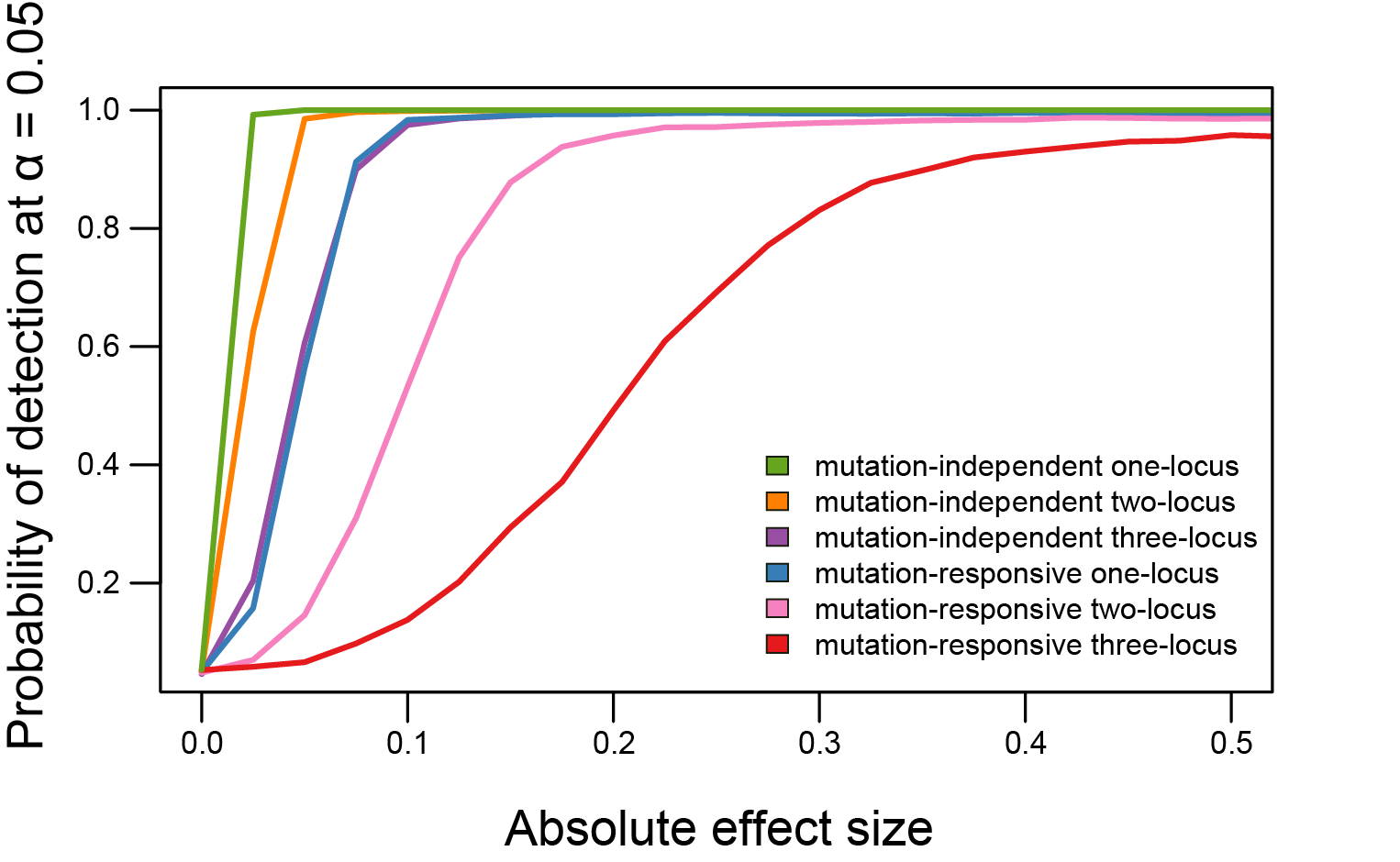
**

**Supplementary Figure 7. Statistical power analysis for one, two, and three-locus interactions.** We determined our statistical power to detect different types of mutation-independent and mutation-responsive genetic effects. This was done by simulating phenotype data for the 1,411 segregants and then performing genetic mapping on the simulated phenotype data (**Methods**). Each set of simulated phenotypes was determined based on which background of the BYx3S cross a segregant came from, as well as the segregant’s genotype at one or more randomly chosen loci, and a knockout that was randomly selected to interact with the loci. We then applied a random deviate to each segregant’s phenotype, which was intended to represent environmental noise. The relevant fixed-effects linear model was fit using the genotype and simulated phenotype data, and the p-value for the appropriate term in the model was obtained. Statistical power for a given absolute effect size was calculated as the proportion of tests that had a p-value ≤ 0.05. These simulations are based on our real phenotype data for glucose. In this environment, we detected average absolute effect sizes of 0.07, 0.15, and 0.3 for mutation-independent one, two-, and three-locus effects, and 0.09, 0.13, and 0.26 for mutation-responsive one-, two-, and three-locus effects.


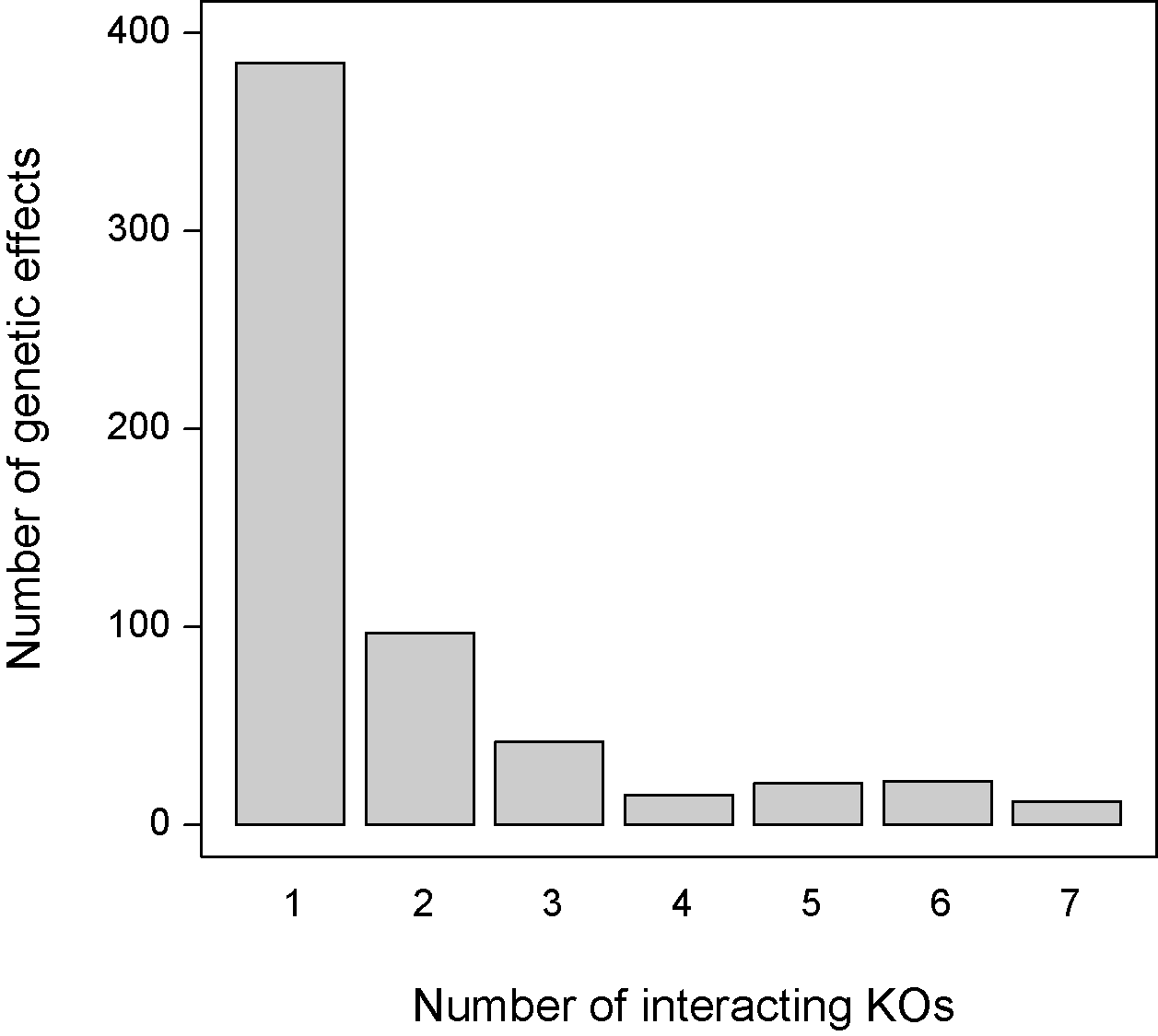


**Supplementary Figure 8. Extent to which mutation-responsive effects interact with different knockouts.** The number of knockout backgrounds in which a particular mutation-responsive effect was detected is shown on the x-axis. The number of mutation-responsive effects in each class is shown on the y-axis.


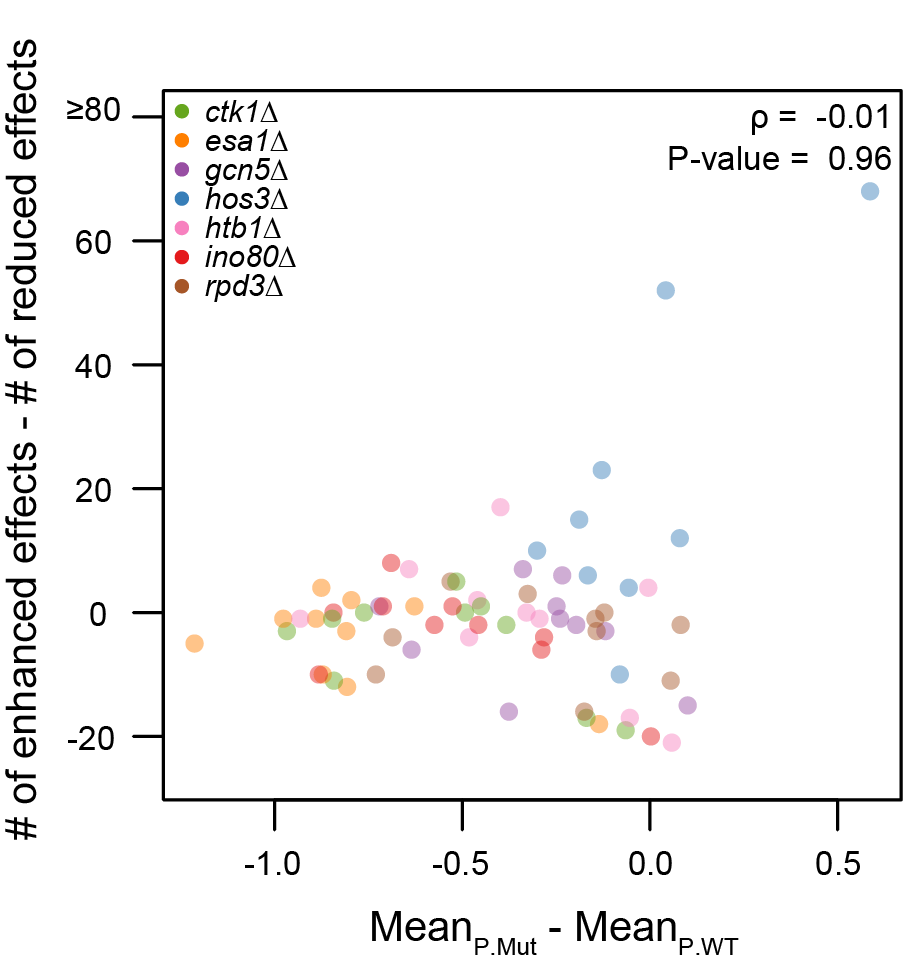


**Supplementary Figure 9. Absence of a relationship between identified mutation-responsive effects and mean phenotypic differences between knockout and wild type backgrounds.** Each point represents a different knockout background and environment. A point’s position on the x-axis indicates the difference in mean between a particular knockout (Mean_P.Mut_) background and the wild type (Mean_P.WT_) background in a single environment. On the y-axis, the difference in the number of genetic effects with enhanced and reduced phenotypic effect in mutants relative to wild type segregants are shown. The spearman’s ρ and its associated p-value are provided on the plot.


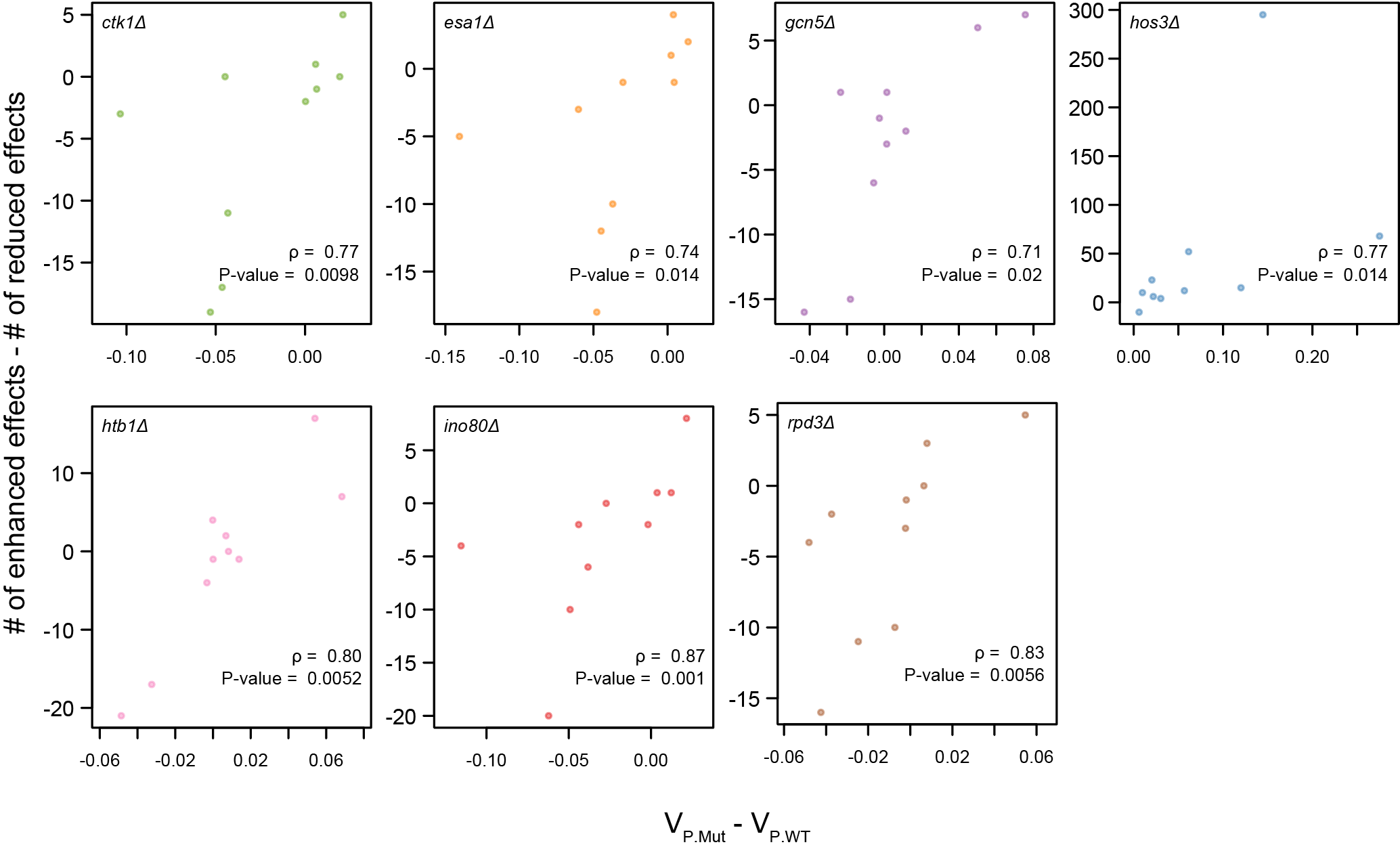


**Supplementary Figure 10. All seven knockout populations show nominally significant correlations between changes in phenotypic variance and detected mutation-responsive effects.** Individual panels show the results for the *ctk1∆*, *esa1∆*, *gcn5∆*, *hos3∆*, *htb1∆*, *ino80∆*, and *rpd3∆* backgrounds. Each point’s position on the x-axis represents the difference in phenotypic variance between wild type and knockout populations. On the y-axis, the difference in the number of genetic effects with enhanced and reduced phenotypic effect in mutants relative to wild type segregants are shown. The spearman’s ρ values and their associated p-values are provided on the plot.


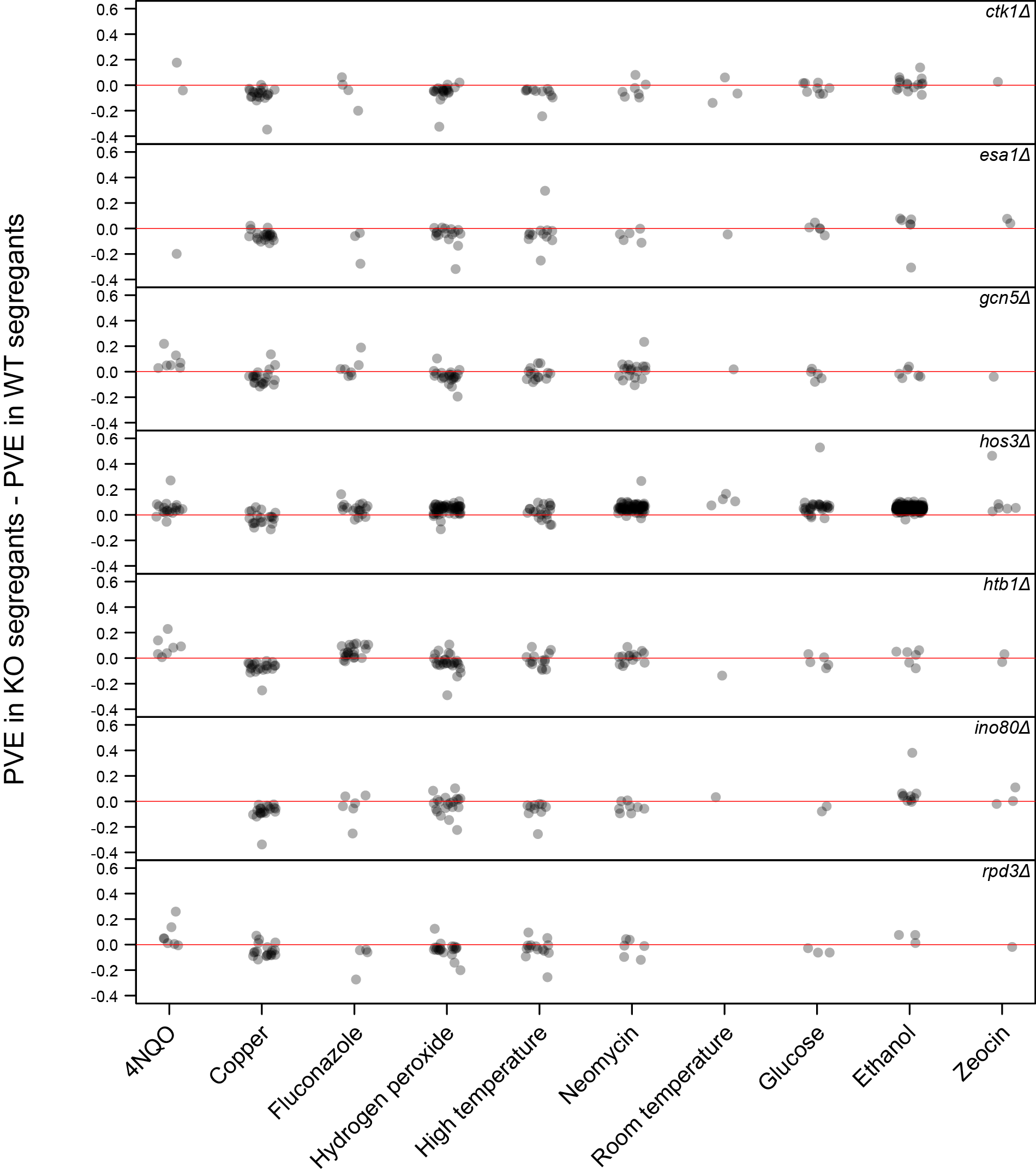


**Supplementary Figure 11. Most mutation-responsive effects show small differences in phenotypic variance explained (PVE) in mutants relative to wild type segregants.** On the y-axis, we show differences in PVE for each mutation-responsive effect when PVE is computed separately in mutant and wild type segregants. Mutation-responsive effects above the red line have a higher PVE in mutants , while mutation-responsive effects below the red line have a higher PVE in wild type segregants.

**Supplementary Tables**

| **Standard Name** | **Systematic Name** | **Function(s)** |
| --- | --- | --- |
| *ASF1* | YJL115W | Nucleosome assembly factor; involved in chromatin assembly, disassembly |
| *CHD1* | YER164W | Chromatin remodeler; regulate chromatin structure and maintain chromatin integrity |
| *CTK1* | YKL139W | Catalytic (alpha) subunit of C-terminal domain kinase I (CTDK-I); required for H3K36 trimethylation but not dimethylation by Set2p |
| *DOT1* | YDR440W | Nucleosomal histone H3-Lys79 methylase |
| *EAF3* | YPR023C | Subunit of Rpd3S deacetylase and NuA4 acetyltransferase complexes; essential histone acetyltransferase complex |
| *ELP3* | YPL086C | Subunit of Elongator complex; exhibits histone acetyltransferase activity |
| *ESA1* | YOR244W | Catalytic subunit of the histone acetyltransferase complex (NuA4) |
| *GCN5* | YGR252W | Catalytic subunit of ADA and SAGA histone acetyltransferase complexes |
| *GIS1* | YDR096W | Histone demethylase and transcription factor |
| *HAT1* | YPL001W | Catalytic subunit of the Hat1p-Hat2p histone acetyltransferase complex |
| *HAT2* | YEL056W | Subunit of the Hat1p-Hat2p histone acetyltransferase complex |
| *HDA1* | YNL021W | Putative catalytic subunit of a class II histone deacetylase complex |
| *HHF1* | YBR009C | Histone H4 |
| *HHF2* | YNL030W | Histone H4 |
| *HHO1* | YPL127C | Histone H1 |
| *HHT1* | YBR010W | Histone H3 |
| *HHT2* | YNL031C | Histone H3 |
| *HIR1* | YBL008W | Subunit of HIR nucleosome assembly complex |
| *HMT1* | YBR034C | Nuclear SAM-dependent mono- and asymmetric methyltransferase |
| *HOS1* | YPR068C | Class I histone deacetylase (HDAC) family member |
| *HOS2* | YGL194C | Histone deacetylase and subunit of Set3 and Rpd3L complexes |
| *HOS3* | YPL116W | Trichostatin A-insensitive homodimeric histone deacetylase (HDAC) |
| *HST1* | YOL068C | NAD(+)-dependent histone deacetylase |
| *HTA1* | YDR225W | Histone H2A |
| *HTA2* | YBL003C | Histone H2A |
| *HTB1* | YDR224C | Histone H2B |
| *HTB2* | YBL002W | Histone H2B |
| *HTZ1* | YOL012C | Histone variant H2AZ |
| *INO80* | YGL150C | ATPase and nucleosome spacing factor |
| *ISW1* | YBR245C | ATPase subunit of imitation-switch (ISWI) class chromatin remodelers |
| *JHD1* | YER051W | JmjC domain family histone demethylase specific for H3-K36 |
| *JHD2* | YJR119C | JmjC domain family histone demethylase |
| *NAT4* | YMR069W | N alpha-acetyl-transferase |
| *RLF2* | YPR018W | Largest subunit (p90) of the Chromatin Assembly Complex (CAF-1) |
| *RPD3* | YNL330C | Histone deacetylase, component of both the Rpd3S and Rpd3L complexes |
| *RPH1* | YER169W | JmjC domain-containing histone demethylase |
| *RTT109* | YLL002W | Histone acetyltransferase |
| *SAS2* | YMR127C | Histone acetyltransferase (HAT) catalytic subunit of the SAS complex |
| *SAS3* | YBL052C | Histone acetyltransferase catalytic subunit of NuA3 complex |
| *SET1* | YHR119W | Histone methyltransferase, subunit of the COMPASS (Set1C) complex |
| *SET2* | YJL168C | Histone methyltransferase with a role in transcriptional elongation |
| *SET3* | YKR029C | Subunit of the *SET3* histone deacetylase complex |
| *SET4* | YJL105W | Unknown function; paralog of *SET3* |
| *SNF1* | YDR477W | AMP-activated S/T protein kinase; regulates H3 acetylation and chromatin remodelling |
| *SNF2* | YOR290C | Catalytic subunit of the SWI/SNF chromatin remodeling complex |
| *SNF5* | YBR289W | Subunit of the SWI/SNF chromatin remodeling complex |
| *SWR1* | YDR334W | Swi2/Snf2-related ATPase; structural component of the SWR1 complex, which exchanges histone variant H2AZ (Htz1p) for chromatin-bound histone H2A |

**Supplementary Table 1. List of 47 genes that were screened for background effects.** The standard gene name, the systematic name, and the biological function of each gene are shown.

| **Knockout** | **Mean Growth** | **Variance** | **Levene's p-value** |
| --- | --- | --- | --- |
| *asf1Δ* | 0.779317208 | 0.020201882 | 0.977508865 |
| *chd1Δ* | 1.007573633 | 0.008422714 | 0.012616542 |
| *ctk1Δ* | 0.505330301 | 0.046562534 | 0.014559018 |
| *dot1Δ* | 1.000469872 | 0.026939673 | 0.572692518 |
| *eaf3Δ* | 0.892511372 | 0.02996817 | 0.159451789 |
| *elp3Δ* | 0.938803654 | 0.020439982 | 0.89517808 |
| *esa1Δ* | 0.299208796 | 0.124484989 | 0.009723665 |
| *gcn5Δ* | 0.727106888 | 0.050249092 | 0.033325098 |
| *gis1Δ* | 1.062032492 | 0.012881687 | 0.134256473 |
| *hat1Δ* | 1.076895249 | 0.027302896 | 0.235343973 |
| *hat2Δ* | 1.051544847 | 0.023497867 | 0.757295234 |
| *hda1Δ* | 0.932223832 | 0.029161218 | 0.50857877 |
| *hhf1Δ* | 0.991287515 | 0.013080162 | 0.102604216 |
| *hhf2Δ* | 0.957913123 | 0.012705815 | 0.123495677 |
| *hho1Δ* | 1.014461673 | 0.013516731 | 0.147089216 |
| *hht1Δ* | 1.007595814 | 0.027733915 | 0.488955838 |
| *hht2Δ* | 1.021186261 | 0.020401193 | 0.968439543 |
| *hir1Δ* | 0.903639888 | 0.022913698 | 0.783955806 |
| *hmt1Δ* | 0.958772913 | 0.021849944 | 0.591705973 |
| *hos1Δ* | 0.947034124 | 0.047755186 | 0.118881424 |
| *hos2Δ* | 0.979073108 | 0.015371335 | 0.275248056 |
| *hos3Δ* | 0.882145103 | 0.07291512 | 2.51478E-05 |
| *hst1Δ* | 0.859791025 | 0.028231512 | 0.552051447 |
| *hta1Δ* | 0.955155532 | 0.032539889 | 0.213817393 |
| *hta2Δ* | 1.051833126 | 0.042651912 | 0.125633861 |
| *htb1Δ* | 0.453580596 | 0.070488173 | 0.002211331 |
| *htb2Δ* | 1.058080223 | 0.009848719 | 0.035718658 |
| *htz1Δ* | 0.739161229 | 0.016096516 | 0.299152086 |
| *ino80Δ* | 0.368393318 | 0.068870748 | 0.000178763 |
| *isw1Δ* | 0.999762052 | 0.018146537 | 0.389392505 |
| *jhd1Δ* | 0.985389791 | 0.018189507 | 0.863810698 |
| *jhd2Δ* | 1.015143929 | 0.028568957 | 0.436252639 |
| *nat4Δ* | 1.007693438 | 0.024951602 | 0.653665809 |
| *rlf2Δ* | 0.992563075 | 0.040109928 | 0.850565985 |
| *rpd3Δ* | 0.733300085 | 0.023198875 | 0.657552748 |
| *rph1Δ* | 0.977926455 | 0.022801194 | 0.949320021 |
| *rtt109Δ* | 0.787318686 | 0.014075713 | 0.210182586 |
| *sas2Δ* | 1.032806204 | 0.015742986 | 0.32742934 |
| *sas3Δ* | 0.98983123 | 0.017062259 | 0.448430416 |
| *set1Δ* | 0.963951523 | 0.045359724 | 0.021614959 |
| *set2Δ* | 0.917625313 | 0.02221723 | 0.963192992 |
| *set3Δ* | 1.011235345 | 0.011976087 | 0.092060159 |
| *set4Δ* | 1.042182908 | 0.022035319 | 0.910385044 |
| *snf1Δ* | 0.236803736 | 0.011258233 | 0.0814094 |
| *snf2Δ* | 0.172978254 | 0.019080041 | 0.238953594 |
| *snf5Δ* | 0.315511891 | 0.012033825 | 0.1426345 |
| *swr1Δ* | 0.817082916 | 0.023596511 | 0.810466613 |
| WT | 1.000603396 | 0.024287414 | NA |

**Supplementary Table 2. Screen summary statistics**. The mean growth and variance for each of the 48 knockout and wild type segregant backgrounds are listed in this table. The Levene’s p-value column specifies the p-value statistic from the Levene’s test used to assess whether the populations of knockout segregants exhibited significantly different heritable phenotypic variation than a wild type BYx3S segregant population on ethanol.

| **Population** | **Number of segregants with good data** | **Diploid** | **Aneuploid** | **Low coverage, cross-contamination** |
| --- | --- | --- | --- | --- |
| WT | 164 | 0 | 0 | 76 |
| *ctk1Δ* | 210 | 0 | 2 | 28 |
| *esa1Δ* | 122 | 0 | 25 | 93 |
| *gcn5Δ* | 215 | 9 | 15 | 1 |
| *hos3Δ* | 220 | 1 | 3 | 16 |
| *htb1Δ* | 177 | 1 | 2 | 60 |
| *ino80Δ* | 141 | 5 | 5 | 89 |
| *rpd3Δ* | 162 | 0 | 0 | 78 |
| **Total** | **1411** | **16** | **52** | **441** |

**Supplementary Table 3. Mapping population breakdown**. This table shows the number of segregants from each knockout populations that were excluded due to technical issues (i.e., low coverage or cross-contamination) or biological issues (i.e., anueploidy or diploidy).

| **Chromosome** | **Start position** | **End position** |
| --- | --- | --- |
| 4 | 720061 | 861257 |
| 4 | 975252 | 1408452 |
| 5 | 190283 | 256159 |
| 7 | 84678 | 170211 |
| 7 | 275701 | 386593 |
| 7 | 905443 | 946974 |
| 10 | 642317 | 684208 |
| 11 | 53931 | 316100 |
| 12 | 50192 | 749934 |
| 13 | 75046 | 130721 |
| 14 | 231034 | 607186 |
| 15 | 69484 | 462566 |
| 15 | 843892 | 959820 |
| 16 | 195879 | 525073 |

**Supplementary Table 4. Genomic regions with allele frequency bias.**  Regions on the genome where the allele frequency in at least one knockout population significantly differed from other populations were found using chi-square tests. Specifically, 2x8 contingency tables were generated and chi-square tests were ran for all 8,311 markers, counting the number of BY and 3S allele in the wild type and 7 knockout populations. Bonferroni correction was used for multiple testing correction.

| **Hemizygote** | **Chromosome** | **Start Position** | **Stop Position** | **Range (bp)** | **Fixed Allele** | **Positions confirmed by Sanger Sequencing** |
| --- | --- | --- | --- | --- | --- | --- |
| *ctk1∆/CTK1* | 11 | 0 | 182309 | 182309 | BY | 85326 - 85727, 118436 - 119014, and 178304 - 178885 |
| *esa1∆/ESA1* | 15 | 657980 | 663812 | 5832 | 3S | 658744 - 659697 |
| *gcn5∆/GCN5* | 4 | 969630 | 985630 | 11205 | 3S | 975757 - 976743 |
| *gcn5∆/GCN5* | 4 | 1123442 | 1408596 | 285154 | 3S | 1140174 - 1140998, 1251735 - 1252459, and 1386674 - 1387285 |
| *gcn5∆/GCN5* | 4 | 1483478 | 1525043 | 41565 | 3S | 1491421 - 1492191 |
| *gcn5∆/GCN5* | 12 | 519517 | 543736 | 24219 | BY | 529628 - 530346 |
| *hos3∆/HOS3* | 13 | 524221 | 526917 | 2696 | 3S | 524221 - 524865 |

**Supplementary Table 5. Fixed regions in BY/3S hemizygous diploids.** This summary table shows regions in the genome where all segregants within a knockout segregant population carried the same parental haplotype due to mitotic recombination in our parental diploids. Also listed are positions within the fixed regions that were PCRed and Sanger sequenced to confirm that these regions were fixed in the parental diploids.

| **Condition** | **Stock solution** | **Amount added per liter** | **Final Concentration** |
| --- | --- | --- | --- |
| 4NQO | 0.5 mg/ml | 500 uL | .25ug/ml |
| Copper | 500mM | 18 mL | 9 mM |
| Ethanol | 20% | 100mL | 2% Ethanol |
| Fluconazole | 10 mg/ml | 5 mL | 50 ug/ml |
| Glucose | NA | NA | 2% Glucose |
| High temperature | NA | NA | 2% Glucose/42°C |
| Hydrogen peroxide | 30% | 541uL | 260 uM |
| Neomycin | 50 mg/ml | 140 mL | 7 mg/ml |
| Room temperature | NA | NA | 2% Glucose/22°C |
| Zeocin | 100 mg/ml | 50 uL | 5 ug/ml |

**Supplementary Table 6. Phenotyping environments.** This table provides information on the environments used for phenotyping assays in our study. Drugs and chemicals were added to 2% glucose plates with the exception of the ethanol condition, in which 2% ethanol was used as the carbon source instead of glucose.

|  | **# of Mutation-independent effects** | | **# of Mutation-responsive effects** | |
| --- | --- | --- | --- | --- |
|  | **Two-locus** | **Three-locus** | **Two-locus** | **Three-locus** |
| **α 0.05** | 17 | 19 | 295 | 670 |
| **FDR 0.10** | 17 | 2 | 542 | 2324 |
| **FDR 0.05** | 10 | 1 | 250 | 1311 |
| **FDR 0.01** | 4 | 0 | 79 | 389 |

**Supplementary Table 7. Number of mutation-independent and mutation-responsive genetic effects across different significance thresholds.** One-locus effects are excluded from this analysis as we only performed scans for these effects at a commonly used significance threshold of α=0.05. Total numbers of all identified genetic effects, including one-locus effects, can be found in **Supplementary Table 9**.

|  | **α 0.05** | | **FDR 0.01** | | **FDR 0.05** | | **FDR 0.10** | | **α 0.05 filtered** | |
| --- | --- | --- | --- | --- | --- | --- | --- | --- | --- | --- |
| **No. interacting KOs** | PVE_KO_ > PVE_WT_ | PVE_KO_ < PVE_WT_ | PVE_KO_ > PVE_WT_ | PVE_KO_ < PVE_WT_ | PVE_KO_ > PVE_WT_ | PVE_KO_ < PVE_WT_ | PVE_KO_ > PVE_WT_ | PVE_KO_ < PVE_WT_ | PVE_KO_ > PVE_WT_ | PVE_KO_ < PVE_WT_ |
| **1** | 383 | 2 | 303 | 3 | 893 | 7 | 1485 | 16 | 178 | 0 |
| **2** | 174 | 20 | 92 | 8 | 274 | 48 | 478 | 92 | 70 | 8 |
| **3** | 76 | 50 | 37 | 26 | 97 | 59 | 142 | 110 | 34 | 32 |
| **4** | 9 | 51 | 3 | 21 | 15 | 69 | 35 | 133 | 3 | 21 |
| **5** | 21 | 84 | 13 | 27 | 20 | 80 | 28 | 157 | 3 | 32 |
| **6** | 12 | 120 | 6 | 36 | 6 | 72 | 11 | 181 | 3 | 57 |
| **7** | 3 | 81 | 1 | 13 | 3 | 39 | 3 | 116 | 2 | 40 |
| **Χ^2^** | 709.37 | | 343.92 | | 981.52 | | 1870.9 | | 341.41 | |
| **p-value** | 5.81E-150 | | 3.12E-71 | | 8.87E-209 | | < 2.2E-16 (0) | | 1.08E-70 | |

**Supplementary Table 8. Chi-squared test results for mutation-responsive effects.**This table shows p-values from chi-squared tests used to test if the ratio of mutation-responsive genetic effects with enhanced and reduced phenotypic effects in mutants changes as a function of number of knockouts a loci interacts (**Methods**). Results are reported across significance thresholds, as well as at the initial α = 0.05 threshold after filtering out effects involving loci with biased individual or multi-locus allele frequences. Test statistics are also reported (Χ-sq statistics).

| **Condition** | **α 0.05** | **FDR 0.01** | **FDR 0.05** | **FDR 0.10** | **α 0.05 filtered** |
| --- | --- | --- | --- | --- | --- |
| 4-NQO | 0.070 | 0.167 | 0.154 | 0.118 | 0.000 |
| Copper | 0.104 | 0.148 | 0.118 | 0.084 | 0.123 |
| Fluconazole | 0.270 | 0.846 | 0.591 | 0.286 | 0.053 |
| Hydrogen peroxide | 0.136 | 0.183 | 0.109 | 0.100 | 0.115 |
| High temperature | 0.189 | 0.400 | 0.400 | 0.147 | 0.179 |
| Neomycin | 0.262 | 0.391 | 0.204 | 0.175 | 0.175 |
| Room temperature | 0.364 | 0.500 | 0.500 | 0.367 | 0.000 |
| Glucose | 0.649 | 0.769 | 0.645 | 0.635 | 0.586 |
| Ethanol | 0.437 | 0.443 | 0.331 | 0.293 | 0.405 |
| Zeocin | 0.500 | 0.875 | 0.875 | 0.875 | 0.500 |
| Average | 0.298 | 0.472 | 0.393 | 0.308 | 0.214 |

**Supplementary Table 9. Percentage of mutation-responsive genetic effects that showed phenotypic effect in any environment outside the one in which they were originally detected.**  Results are reported across significance thresholds, as well as at the initial α = 0.05 threshold after filtering out effects involving loci with biased individual or multi-locus allele frequences on different tabs.

|  | **1-locus interaction** | **2-locus interaction** | **3-locus interaction** |
| --- | --- | --- | --- |
| Mutation-responsive without filtering | 45 | 143 | 406 |
| Mutation-responsive with filtering | 14 | 73 | 181 |
| Mutation-independent | 89 | 17 | 19 |

**Supplementary Table 10. Number of mutation-responsive effects that show biased individual and multi-locus allele frequencies.** The numbers of two-locus and three-locus mutation-responsive effects that show biased individual and multi-locus allele frequencies were determined using chi-square tests. Specifically, 2x8, 4x8, and 8x8 contingency tables were generated and chi-square tests were ran for all one, two, and three-locus interactions respectively, counting the number of each loci combination in the wild type and 7 knockout populations. Bonferroni correction was used for multiple testing correction.

|  | **4NQO** | **Copper** | **Flu** | **H2O2** | **HT** | **Neo** | **RT** | **Glu** | **Eth** | **Zeo** |
| --- | --- | --- | --- | --- | --- | --- | --- | --- | --- | --- |
| ctk1.Enh | 1 | 1 | 2 | 2 | 0 | 2 | 1 | 3 | 10 | 1 |
| ctk1.Red | 1 | 20 | 2 | 19 | 11 | 5 | 2 | 5 | 5 | 0 |
| ctk1.Diff | 0 | -19 | 0 | -17 | -11 | -3 | -1 | -2 | 5 | 1 |
| esa1.Enh | 0 | 2 | 0 | 2 | 1 | 0 | 0 | 3 | 5 | 2 |
| esa1.Red | 1 | 20 | 3 | 14 | 11 | 5 | 1 | 2 | 1 | 0 |
| esa1.Diff | -1 | -18 | -3 | -12 | -10 | -5 | -1 | 1 | 4 | 2 |
| gcn5.Enh | 7 | 3 | 4 | 3 | 4 | 12 | 1 | 1 | 2 | 0 |
| gcn5.Red | 0 | 18 | 3 | 19 | 10 | 6 | 0 | 4 | 4 | 1 |
| gcn5.Diff | 7 | -15 | 1 | -16 | -6 | 6 | 1 | -3 | -2 | -1 |
| hos3.Enh | 17 | 5 | 15 | 55 | 15 | 70 | 4 | 26 | 296 | 6 |
| hos3.Red | 2 | 15 | 3 | 3 | 5 | 2 | 0 | 3 | 1 | 0 |
| hos3.Diff | 15 | -10 | 12 | 52 | 10 | 68 | 4 | 23 | 295 | 6 |
| htb1.Enh | 7 | 0 | 19 | 5 | 5 | 9 | 0 | 2 | 4 | 1 |
| htb1.Red | 0 | 21 | 2 | 22 | 9 | 5 | 1 | 3 | 2 | 1 |
| htb1.Diff | 7 | -21 | 17 | -17 | -4 | 4 | -1 | -1 | 2 | 0 |
| ino80.Enh | 0 | 0 | 2 | 7 | 0 | 2 | 1 | 0 | 9 | 2 |
| ino80.Red | 0 | 20 | 4 | 13 | 10 | 6 | 0 | 2 | 1 | 1 |
| ino80.Diff | 0 | -20 | -2 | -6 | -10 | -4 | 1 | -2 | 8 | 1 |
| rpd3.Enh | 6 | 4 | 0 | 2 | 2 | 2 | 0 | 0 | 3 | 0 |
| rpd3.Red | 1 | 15 | 4 | 18 | 12 | 4 | 0 | 3 | 0 | 1 |
| rpd3.Diff | 5 | -11 | -4 | -16 | -10 | -2 | 0 | -3 | 3 | -1 |
| WT.Mean | 0.918 | 0.436 | 0.875 | 0.896 | 0.896 | 1.374 | 1.053 | 1.016 | 1.020 | 0.973 |
| ctk1.Mean | 0.425 | 0.372 | 0.113 | 0.727 | 0.054 | 0.407 | 0.206 | 0.633 | 0.504 | 0.522 |
| esa1.Mean | 0.028 | 0.301 | 0.066 | 0.089 | 0.024 | 0.161 | 0.076 | 0.388 | 0.144 | 0.177 |
| gcn5.Mean | 0.579 | 0.537 | 0.154 | 0.521 | 0.260 | 1.141 | 0.804 | 0.897 | 0.824 | 0.733 |
| hos3.Mean | 0.729 | 0.356 | 0.954 | 0.939 | 0.595 | 1.961 | 0.996 | 0.887 | 0.793 | 0.807 |
| htb1.Mean | 0.276 | 0.495 | 0.476 | 0.843 | 0.414 | 1.370 | 0.121 | 0.721 | 0.560 | 0.644 |
| ino80.Mean | 0.075 | 0.439 | 0.300 | 0.607 | 0.014 | 1.092 | 0.341 | 0.558 | 0.330 | 0.447 |
| rpd3.Mean | 0.387 | 0.492 | 0.189 | 0.721 | 0.166 | 1.456 | 0.931 | 0.874 | 0.694 | 0.827 |
| WT.Var | 0.046 | 0.077 | 0.083 | 0.074 | 0.050 | 0.183 | 0.012 | 0.012 | 0.022 | 0.014 |
| ctk1.Var | 0.065 | 0.024 | 0.039 | 0.028 | 0.007 | 0.080 | 0.018 | 0.012 | 0.043 | 0.020 |
| esa1.Var | 0.015 | 0.029 | 0.023 | 0.029 | 0.013 | 0.043 | 0.016 | 0.014 | 0.026 | 0.028 |
| gcn5.Var | 0.121 | 0.059 | 0.060 | 0.031 | 0.044 | 0.233 | 0.013 | 0.013 | 0.033 | 0.011 |
| hos3.Var | 0.166 | 0.083 | 0.140 | 0.136 | 0.060 | 0.459 | 0.042 | 0.032 | 0.166 | 0.036 |
| htb1.Var | 0.114 | 0.028 | 0.137 | 0.042 | 0.047 | 0.183 | 0.026 | 0.012 | 0.029 | 0.022 |
| ino80.Var | 0.018 | 0.015 | 0.039 | 0.036 | 0.001 | 0.068 | 0.024 | 0.010 | 0.043 | 0.017 |
| rpd3.Var | 0.100 | 0.052 | 0.035 | 0.032 | 0.043 | 0.146 | 0.018 | 0.009 | 0.030 | 0.012 |

**Supplementary Table 11. Phenotypic variance and the number of identified genetic effects**. This is a summary table showing the phenotypic mean and variance in the *ctk1Δ*, *esa1Δ*, *gcn5Δ*, *hos3Δ*, *htb1Δ*, *ino80Δ*, *rpd3Δ,* and wild type populations across 10 environments. In addition, the numbers of genetic effects with reduced (Red) and enhanced (Enh) effects in the knockout background in which the genetic effect was identified relative to wild type background are shown.

| **Primer Name** | **Primer Sequence** |
| --- | --- |
| ASF1 Diagnostic F | GTGCCACACCTAACCTTCGA |
| ASF1 Diagnostic R | CCGCATCCTTTGGAGTGGAT |
| ASF1 MX F | CTCGAAAGTGTAACAGCGTACTCTCCCTACCATCCAATTGAAACATAAGATATAGAAAAGCCTTGACAGTCTTGACGTGC |
| ASF1 MX R | ATACATTTTATAAAGTGTACCTCTCTTGCAGGTACCATTAATCTTATAACCCATAAATTCCGCACTTAACTTCGCATCTG |
| CHD1 Diagnostic F | CCTCAGCCCATCAATGCGTA |
| CHD1 Diagnostic R | GTATCCAACCAGGCAGGCAT |
| CHD1 MX F | ATTTCTTTAAACCTATACCCAATTCAAAGCAGAACCTTTTCTAATTTAATTCTCACTTAT CCTTGACAGTCTTGACGTGC |
| CHD1 MX R | AATACGTTTATAGTTATGGGGGGAAGGAACAATGGAAAATGTGGTGAAGAAAAATTGTTT CGCACTTAACTTCGCATCTG |
| CTK1 Diagnostic F | CAACTCTTCGACAACTGCGC |
| CTK1 Diagnostic R | TGCTCCTTTGCTGCGTTAGA |
| CTK1 MX F | AGCACTATTCTTTGCACTAGAATAACACAGGGACCATACAGCATAAATTATTTGGTAACACCTTGACAGTCTTGACGTGC |
| CTK1 MX R | GTAATAAATAAGTTATTAATCTATTTTTTGTGTCTACTTATTTCAATTGGCTATATATCCCGCACTTAACTTCGCATCTG |
| CYC8 Diagnostic F | TCCACCGTAGAACCCAAAGC |
| CYC8 Diagnostic R | TAATTGGCGCACAGGAACCT |
| CYC8 MX F | GCTACACAACATTTCTCGTTGATTATAAATTAGTAGATTAATTTTTTGAATGCAAACTTTCCTTGACAGTCTTGACGTGC |
| CYC8 MX R | TACAACTACAACAGCAACAACAACAAACAAAACACGACTGGAAAAAAAAAATTAGGAAAACGCACTTAACTTCGCATCTG |
| DOT1 Diagnostic F | TGGATGAAAGAGCTCTGGCA |
| DOT1 Diagnostic R | AGACCAGGCAGCTGTATTCA |
| DOT1 MX F | AATGGGCGGTCAAGAAAGTATATCAAATAATAACTCAGACTCATTCATTATGTCGTCCCCCCTTGACAGTCTTGACGTGC |
| DOT1 MX R | GTGTACATGTTATTTCTACTTAGTTATTCATACTCATCGTTAAAAGCCGTTCAAAGTGCCCGCACTTAACTTCGCATCTG |
| EAF3 Diagnostic F | TTGCTGCGTCAGAGGGTTAA |
| EAF3 Diagnostic R | TTGCTGCGTCAGAGGGTTAA |
| EAF3 MX F | ATTGCATAAATACGGAAGAACTAAATACTAGAAATAATCCCAAGCTAGAATATAAACGTCCCTTGACAGTCTTGACGTGC |
| EAF3 MX R | CATTTTGCATTAGCATCTGTGAGGCCTCGTCACTGGATTTACCCTATTGAAGAACGTATACGCACTTAACTTCGCATCTG |
| ELP3 Diagnostic F | ATCCAAATGACTTCTTATTT |
| ELP3 Diagnostic R | TATACAGCGATAAGACAGTG |
| ELP3 MX F | ATTTAAATTTCTGCTTGGAAAACCGGCCATGTCGGCGGCACATAAAAGTTCTATTTACCT CCTTGACAGTCTTGACGTGC |
| ELP3 MX R | GTTTTGAAATAAACAAGTCCTAAAAGCACCTAAGGAAAATCGAAGAACACCCTGACAAAG CGCACTTAACTTCGCATCTG |
| ESA1 Diagnostic F | GGCCTCTAAATCCTGGCAAT |
| ESA1 Diagnostic R | AGTGCCTGTGTTTGCATTTG |
| ESA1 MX F | TTACCATTCTTTAGACGCTTCCTGTGCTACCATTCTCGGAAATACTGCAAGAAATCATCG CCTTGACAGTCTTGACGTGC |
| ESA1 MX R | TATTTAAAGCTTTTACATTAGAAGTTGTTTGAATGTAAGTTTAGGAAAGCACTACATAGC CGCACTTAACTTCGCATCTG |
| GCN5 Diagnostic F | GCACACAAGATGACGCTTCC |
| GCN5 Diagnostic R | CTCGCCATTGTACATTCGGC |
| GCN5 MX F | AAGGGAAGACCGTGAGCCGCCCAAAAGTCTTCAGTTAACTCAGGTTCGTATTCTACATTA CCTTGACAGTCTTGACGTGC |
| GCN5 MX R | CGTACTAAACATTTATTTCTTCTTCGAAAGGAATAGTAGCGGAAAAGCTTCTTCTACGCA CGCACTTAACTTCGCATCTG |
| GIS1 Diagnostic F | TGGCGTTTTGTGCTTCAAGG |
| GIS1 Diagnostic R | TGTTTGCGGACGGTATGGAA |
| GIS1 MX F | AACAACATCGTTGTAATTTTTTTTTTTTAATTTGAAGAATAGCTACAAAAACAGACTACACCTTGACAGTCTTGACGTGC |
| GIS1 MX R | TACAGGAAAATATTCGATAAAAATTTTTTTTGAACCCATTTTGTATATCATTTTCTTGACCGCACTTAACTTCGCATCTG |
| HAT1 Diagnostic F | CAAACTGTTTCATGTTAGTG |
| HAT1 Diagnostic R | TAAATTCTTGATCAAATACG |
| HAT1 MX F | TTCTGGAATTGTTTTCAGCAAAATTATGCTTAAGCTATAACTATAGTGAGAATCAAGAAT CCTTGACAGTCTTGACGTGC |
| HAT1 MX R | GAATTTCTTATTTCAGGCTTGTTAAACAAATAAATATGTTATTATATATTTAATAAACAG CGCACTTAACTTCGCATCTG |
| HAT2 Diagnostic F | CTCTTTAACGGCGCCTCAAG |
| HAT2 Diagnostic R | CACGATGGGTAGACTATGGGA |
| HAT2 MX F | TATCTCTCCTATCAATTGTGGTTAGCCTAGTAGTCACCAAATAGCAAATTACCAATCAAC CCTTGACAGTCTTGACGTGC |
| HAT2 MX R | TGTATGTTGATCTTTGTTTAATTACGCCTTTTCGCCAAAGAAACAATAAAAAAACTATAT CGCACTTAACTTCGCATCTG |
| HDA1 Diagnostic F | TTATATTTCCAACACGAATCGAGAACT |
| HDA1 Diagnostic F | TTATATTTCCAACACGAATCGAGAACT |
| HDA1 MX F | ACATAACAAAATATTGAGAAAGGGAAAGTTGAGCACTGTAATACGCCGAACAGATTAAGCCCTTGACAGTCTTGACGTGC |
| HDA1 MX R | ATTCAACTTTCATAAGGCATGAAGGTTGCCGAAAAAAAATTATTAATGGCCAGTTTTTCCCGCACTTAACTTCGCATCTG |
| HHF1 Diagnostic F | CAAATTATTCCATCATTAAA |
| HHF1 Diagnostic R | AGTCAAGGAGAGATATTACG |
| HHF1 MX F | CAGTTGAATACGAATCCCAAATATTTGCTTGTTGTTACCGTTTTCTTAGAATTAGCTAAA CCTTGACAGTCTTGACGTGC |
| HHF1 MX R | TGTACTCTATAGTACTAAAGCAACAAACAAAAACAAGCAACAAATATAATATAGTAAAAT CGCACTTAACTTCGCATCTG |
| HHF2 Diagnostic F | AAAAGAACAAGAAAAAGATT |
| HHF2 Diagnostic R | CAACAGATAAATGATGACAA |
| HHF2 MX F | TCTTTTTTCCTACATCTTGTTCAAAAGAGTAGCAAAAACAACAATCAATACAATAAAATA CCTTGACAGTCTTGACGTGC |
| HHF2 MX R | TTTTATTTTTTGAAAGGCATGAAAATAATTTCAAACACCGATTGTTTAACCACCGATTGT CGCACTTAACTTCGCATCTG |
| HHO1 Diagnostic F | CCACGTCGTGAACAGACAGT |
| HHO1 Diagnostic R | GCTGTTTGCTTTGATGAAATGCT |
| HHO1 MX F | AAGAAAATAGGTTTGATAGTATTGCTATCACCATTGACATTCTCGTTTGGATATTCACTT CCTTGACAGTCTTGACGTGC |
| HHO1 MX R | TTATGGGCACCTGATAATGCTTGGCAGCGAGGGAAGCAATTATAATACAACTAAAGCAAC CGCACTTAACTTCGCATCTG |
| HHT1 Diagnostic F | AAGCGCTCGGAACAGTTTTA |
| HHT1 Diagnostic R | GACACCCCAACCTACTCCAA |
| HHT1 MX F | TATATTCTTTCTTTCTAGTTAATAAGAAAAACATCTAACATAAATATATAAACGCAAACA CCTTGACAGTCTTGACGTGC |
| HHT1 MX R | TGATTTATATTTTATTGTGTTTTTGTTCGTTTTTTACTAAAACTGATGACAATCAACAAA CGCACTTAACTTCGCATCTG |
| HHT2 Diagnostic F | TGATTGGTTGTATAAGAAAA |
| HHT2 Diagnostic R | TAAGTAACAGAGTCCCTGAT |
| HHT2 MX F | TGTTTGTATGATGTCCCCCCAGTCTAAATGCATAGAAAAAAAAAAATTCCCGCTTTATAT CCTTGACAGTCTTGACGTGC |
| HHT2 MX R | ACTTTGGCCCTTCCAACTGTTCTTCCCCTTTTACTAAAGGATCCAAGCAAACACTCCACA CGCACTTAACTTCGCATCTG |
| HIR1 Diagnostic F | CGCACTTACTCGATCCTGCT |
| HIR1 Diagnostic R | AGCGGGATCAAAAACAACGC |
| HIR1 MX F | ATGAAAGTGGTAAAGTTTCCATGGTTGGCTCACCGTGAAGAATCGCGAAAATATGAAATACCTTGACAGTCTTGACGTGC |
| HIR1 MX R | ATAAAATATAGACGTAATTATGAGGGAAAAAACTTGTCCAAAGGAAGGGGTATAAGCTTACGCACTTAACTTCGCATCTG |
| HMT1 Diagnostic F | TTGCTGCAATTCGGATGCTG |
| HMT1 Diagnostic R | GACAGCCGTGAAAGATTCTGC |
| HMT1 MX F | TATGAACAAGTTTGTTTATTTGCTTTTCAAATTTTTTTCTTTCTCCAGCAAACAAAAGTC CCTTGACAGTCTTGACGTGC |
| HMT1 MX R | CTGCTCACCTTGCCGTTTCCAAAAAAGAGTTAGAACCGACAAATTCATCCAAAGAAAATA CGCACTTAACTTCGCATCTG |
| HOS1 Diagnostic F | AGCCGTTGATCACACCGAAA |
| HOS1 Diagnostic R | AAACAGAGGGCAAGCGGATT |
| HOS1 MX F | TTACAGTTCGTAAAACTTCATAAGTTCGACCATATCTCTATCCTTATTGTCATTCCTTAACCTTGACAGTCTTGACGTGC |
| HOS1 MX R | AAAAAGGTGTATGTACTGTAATATGAATTAATAAACACCTGTCCATTTTAGAAAAACGCTCGCACTTAACTTCGCATCTG |
| HOS2 Diagnostic F | TCGAATACGGAGTGCACCTT |
| HOS2 Diagnostic R | TCTCGATGTTCTTTGGGGCA |
| HOS2 MX F | GAAAATAAAAAAAAAAAAAAAAAAAAAAACGGGAGATTAACCGAATAGCAAACTCTTAAA CCTTGACAGTCTTGACGTGC |
| HOS2 MX R | AAGACGCCAGATTACTCAAGTACGTTAAAATCAGGTATCAAGTGAATAACAACACGCAAC CGCACTTAACTTCGCATCTG |
| HOS3 Diagnostic F | AGGGAAAAGAATCGGCTGGG |
| HOS3 Diagnostic R | GTCCCATCACCGTGGTGTAG |
| HOS3 MX F | ACTGAAAATATAACGAAAAAAAGGGCTCTGGAAGTAAACAGAGAAATTCGACGATATAATCCTTGACAGTCTTGACGTGC |
| HOS3 MX R | TCACCATCTTCCACCACTTCTTGTTGTATGTTTTCTTGAAACATGAGAAATCATTGATATCGCACTTAACTTCGCATCTG |
| HST1 Diagnostic F | TGGAATCGTTGCTGGGTCAG |
| HST1 Diagnostic R | GCCAGTGGAAGTCAACTCGA |
| HST1 MX F | TTACTGTTGTTTCTTTCGTGGCTGTTTCTTAATCTTATACGTACCTTTATCTATTTCCGT CCTTGACAGTCTTGACGTGC |
| HST1 MX R | TGGTAGTGATACGAACACTTCTCTTCTTTTTTGTTGTTTTTGTGAGAAAAAAAAATCTAA CGCACTTAACTTCGCATCTG |
| HTA1 Diagnostic F | GGTTTCTTTTCAGCTGGGGC |
| HTA1 Diagnostic R | TCAACATGTCACCAGTGGCC |
| HTA1 MX F | TTATTTCTCAGTGAATAAACAACTTCAAAACAAACAAATTTCATACATATAAAATATAAA CCTTGACAGTCTTGACGTGC |
| HTA1 MX R | TAGTTACAATGGAGAAGCAGTTTAGTTCCTTCCGCCTTCTTTAAAATACCAGAACCGATC CGCACTTAACTTCGCATCTG |
| HTA2 Diagnostic F | GCGCCTTCTATTCCGGAGAA |
| HTA2 Diagnostic R | TGACGGCAAGTGTCTCACTG |
| HTA2 MX F | TACTTTAAAACCCCAAATGACAAGAATGTTTGATTTGCTTTGTTTCTTTTCAACTCAGTT CCTTGACAGTCTTGACGTGC |
| HTA2 MX R | ATTCTTGTCTTTTTACATAAGAATTAGGAAAGTACAGAACAAGAGCAAATTTAATATATA CGCACTTAACTTCGCATCTG |
| HTB1 Diagnostic F | ACGATCCAGTCAGCGACATC |
| HTB1 Diagnostic R | AGCCGAAAAGAAACCAGCCT |
| HTB1 MX F | TTAATTTTTATATACCCATATAAATAATAATATTAATTATAACCAAAGGAAGTGATTTCACCTTGACAGTCTTGACGTGC |
| HTB1 MX R | TTATATTAAATTTATCCTATATAGACAAGTCAAACCACAAATAAACCATACACACATACACGCACTTAACTTCGCATCTG |
| HTB2 Diagnostic F | ATGGCCCCCAGGTTAATGTG |
| HTB2 Diagnostic R | ACAGCCCTAGTACCTTCGGA |
| HTB2 MX F | TCTTCTTGTTAATTTTTTCTGATTGCTCTATACTCAAACCAACAACAACTTACTCTACAACCTTGACAGTCTTGACGTGC |
| HTB2 MX R | TATAAAAAATGCCACTAATAAAAAGAAAACATGACTAAATCACAATACCTAGTGAGTGACCGCACTTAACTTCGCATCTG |
| HTZ1 Diagnostic F | CGTTCGTGGAACAGTGAGGA |
| HTZ1 Diagnostic R | GCAACGCACAAAGCTTCGTA |
| HTZ1 MX F | ATAGAATGAGGATACAGGAGCAGGGAGAATTACGGGAAATGGGAAAGAAAAACTATTCTTCCTTGACAGTCTTGACGTGC |
| HTZ1 MX R | GAAAAAATATCGTTAAATTCAATTTCGCACTATAGCCGCACGTAAAAATAACTTAACATACGCACTTAACTTCGCATCTG |
| INO80 Diagnostic F | AGCTTTGGAAAACGGCGAAA |
| INO80 Diagnostic R | ATACTGGATGAGGCCCAAGC |
| INO80 MX F | AGCAGATTAAAGATAGACATTAACTCCGCTTAATGTAAATAACACAATATGAATACCTTTCCTTGACAGTCTTGACGTGC |
| INO80 MX R | ACCGATCCTGTCCATATTAGCAAAGCAAGGCTTAAGACATATAGAAGAGCATTTATAGACCGCACTTAACTTCGCATCTG |
| ISW1 Diagnostic F | CGGCCGGCCATATCTAGAC |
| ISW1 Diagnostic R | CAACCCCACCAAACGTGAAC |
| ISW1 MX F | TTTTCTTCAGAAGCATGGTGTAGGATATATTAAAAAAAATCGAAATATAAAAAAAGAAGG CCTTGACAGTCTTGACGTGC |
| ISW1 MX R | AGAGTCCATATGTATAGCTATGCAAAAACCAGCTAGAGGTGGATGTAGAAATACCCTATT CGCACTTAACTTCGCATCTG |
| JHD1 Diagnostic F | CCTGTGGTGGCATTCATCTTG |
| JHD1 Diagnostic R1 | GTGATTGAACGTCCATTACATCA |
| JHD1 MX F | ATTATAATGAGTAAGAAGACGTAATGATCATAAAACAAAATACTAATAAGCTATGGTGCACCTTGACAGTCTTGACGTGC |
| JHD1 MX R | TTACGAAATAAGATCGGCTAAAGCTTTCGCTAAATGCTTCTTTGAAGTGAAATTTAACGGCGCACTTAACTTCGCATCTG |
| JHD2 Diagnostic F | GTGGAGTTCAAGGCTTGAGC |
| JHD2 Diagnostic R | TGTGCATGTTGACAACGCAG |
| JHD2 MX F | GGCGTGCTAAATGCCAAGTATTATTCTAAAAAATCATTACGCCATACACAAATATTGAAGCCTTGACAGTCTTGACGTGC |
| JHD2 MX R | CAATTTTACCTCTAGATCATATTAACTAATCTCATCTTGCACAAAAAACGTATCACTATCCGCACTTAACTTCGCATCTG |
| KanMX check F | CAGATGCGAAGTTAAGTGCG |
| KanMX check R | GCACGTCAAGACTGTCAAGG |
| NAT4 Diagnostic F | GTATGCCTGGAGTATGGCAGT |
| NAT4 Diagnostic R | AAGCGCCTCATATAGTCGCC |
| NAT4 MX F | CGCGCCATTAAAAGATTTTTTTCTTAGCTCTTTTTCTTTTTTCTTTTTCTTTCCACTGAG CCTTGACAGTCTTGACGTGC |
| NAT4 MX R | GCCGCGTACGTAGTTTTACTTTTAATTTTTTTTTTATCGCGCGTTGTCCCTGTCGGCTTT CGCACTTAACTTCGCATCTG |
| RLF2 Diagnostic F | GCTGGATCAAGTGGTTCCCT |
| RLF2 Diagnostic R | GCACGCCTTTGTCTTTCCTC |
| RLF2 MX F | CAGAGAATTATATGTTTTAGTGAACCTCAAGACAGAAGAGAATCGAAAGGAAAAGGGAAACCTTGACAGTCTTGACGTGC |
| RLF2 MX R | TGTATACCAATAAATAATCAGTTTATCTGTATGTTTCTATATACTAAAGATCCGTTCAAGCGCACTTAACTTCGCATCTG |
| RPD3 Diagnostic F | ACTGGTTTTGTACAGCGCTG |
| RPD3 Diagnostic R | ACTGGTTTTGTACAGCGCTG |
| RPD3 MX F | TTCACTTTTCTTCTTTTGTTTCACATTATTTATATTCGTATATACTTCCAACTCTTTTTTCCTTGACAGTCTTGACGTGC |
| RPD3 MX R | GGTTCATAAAACAATTGCGCCATACAAAACATTCGTGGCTACAACTCGATATCCGTGCAGCGCACTTAACTTCGCATCTG |
| RPH1 Diagnostic F | CATCGCCATGCAATTAATCA |
| RPH1 Diagnostic R | ACCACATGGCAGATGGTTTT |
| RPH1 MX F | AAAAAAAAGGGAAAAAAAGAATAAGACTGTCTTGGTGAGGATATTCAGTTGCGTGAAATC CCTTGACAGTCTTGACGTGC |
| RPH1 MX R | CGAGCACATTTTAAGAGCCTTCAAAATGAGAGATCTCGGTAAACAACTGGCAATCGTGAG CGCACTTAACTTCGCATCTG |
| RSC4 Diagnostic F | CGGAAGACAAGTAGCCCAAGA |
| RSC4 Diagnostic R | CGCCGCTGTAAAACTATCGC |
| RSC4 MX F | ACCCTCAGCCTGTTATTACAATAGAGAGCAAACCAAAGAATAATAGATAAAGTACACAGACCTTGACAGTCTTGACGTGC |
| RSC4 MX R | ATATAGGTTGTATATAGATACATGCATATGATGGGAAGACTATGAAGAGAGAGATAGTCACGCACTTAACTTCGCATCTG |
| RTT109 Diagnostic F | TCGAGGTGTTTCGTCATCGG |
| RTT109 Diagnostic R | GATGTTGCTTGCAGGAACCG |
| RTT109 MX F | TTTGTCAATAGAGTTGTCCAGTAGAGTTAAAAGGTCAATTCAACCGGTCTTCAATAAGAC CCTTGACAGTCTTGACGTGC |
| RTT109 MX R | CATGCATTTTCTAAGATCGATGCTACATACGTGTACTAAATAATAAATATCAATATGTAT CGCACTTAACTTCGCATCTG |
| SAS2 Diagnostic F | CATCGAAAAACCGGCCCAAA |
| SAS2 Diagnostic R | TACACGGATGATCAGACGCG |
| SAS2 MX F | CATCGAGCGATATTCTATCCTGAAATACATATGCCATTAAGTTACATCCTGAATAGATTCCCTTGACAGTCTTGACGTGC |
| SAS2 MX R | ATTTTTTGATATTGGAGGCTCCTATTTTCTAGTTGCTTTTTGTTTTCACTCGCAAAAAAACGCACTTAACTTCGCATCTG |
| SAS3 Diagnostic F | ACCATTTGTCGCCGCTAGAA |
| SAS3 Diagnostic R | TCGCCATAAAACACGCTCAT |
| SAS3 MX F | CTTATTGCTATTAATAATGTTACATGTATATGCTTATATCCAATATATACCCATCGCCGCCCTTGACAGTCTTGACGTGC |
| SAS3 MX R | AAAAGCATTGCTATTCTTTTCTCATAGGTGTTATTCATACCGCCCTCTCTCTTCTTCCTTCGCACTTAACTTCGCATCTG |
| SET1 Diagnostic F | AAATATCCGCTCGACCAGTCC |
| SET1 Diagnostic F | AAATATCCGCTCGACCAGTCC |
| SET1 MX F | CTAGCATAGGTAACATTCCTTATTTGTTGAATCTTTATAAGAGGTCTCTGCGTTTAGAGACCTTGACAGTCTTGACGTGC |
| SET1 MX R | TTTGCTGGAAAGCAACGATATGTTAAATCAGGAAGCTCCAAACAAATCAATGTATCATCGCGCACTTAACTTCGCATCTG |
| SET2 Diagnostic F | ACTAGTCAACGACGCTGACC |
| SET2 Diagnostic R | GCCTGGCGCTTTAGACTCTA |
| SET2 MX F | ACAAGACTTCCTTTGGGACAGAAAACGTGAAACAAGCCCCAAATATGCATGTCTGGTTAACCTTGACAGTCTTGACGTGC |
| SET2 MX R | AAAACTGCATAGTCGTGCTGTCAAACCTTTCTCCTTTCCTGGTTGTTGTTTTACGTGATCCGCACTTAACTTCGCATCTG |
| SET3 Diagnostic F | GCACTAGGCACCAGTGTTGA |
| SET3 Diagnostic R | TCCTTAATCGAAATAATGGTCCAAAA |
| SET3 MX F | TGAATATTCACTTTTGAATATACTTAAGTTTATATAGGTGTAAGAAGGAAATGTCCATGTCCTTGACAGTCTTGACGTGC |
| SET3 MX R | GATTTAAAGCGTATATACAACAGTTTTAGATCGTACTTCACAAAATACGAGAACTGAATCCGCACTTAACTTCGCATCTG |
| SET4 Diagnostic F | ACATGTAGAGGTCACCGGGA |
| SET4 Diagnostic R | GGAGTCACTTGAACCGCAGA |
| SET4 MX F | AACGCCGGAATAAGATTGGTACCCTCGTCAGAAAGTTACAAATACCGCTTCATCTTCAAACCTTGACAGTCTTGACGTGC |
| SET4 MX R | ATGATAAAATTAAGCTTTCAAAAAAGATTAAAATGAATACTATTAATTTTAAAATTTCGTCGCACTTAACTTCGCATCTG |
| SIR2 Diagnostic F | CTTAACACATTTAAACCATG |
| SIR2 Diagnostic R | AGCTCTAATTTGAAAGAAAT |
| SIR2 MX F | TTGCCATACTATGTAAATTGATATTAATTTGGCACTTTTAAATTATTAAATTGCCTTCTA CCTTGACAGTCTTGACGTGC |
| SIR2 MX R | AGGCATCGCTTCGGTAGACACATTCAAACCATTTTTCCCTCATCGGCACATTAAAGCTGG CGCACTTAACTTCGCATCTG |
| SNF1 Diagnostic F | GCAAAAGGATGGGCGTGATG |
| SNF1 Diagnostic F2 | GCAAAAGGATGGGCGTGATG |
| SNF1 MX F | GAAAGAAATAGAAGTTTTTTTTTGTAACAAGTTTTGCTACACTCCCTTAATAAAGTCAACCCTTGACAGTCTTGACGTGC |
| SNF1 MX R | AAATACGTTACGATACATAAAAAAAAGGGAACTTCCATATCATTCTTTTACGTTCCACCACGCACTTAACTTCGCATCTG |
| SNF2 Diagnostic F | TGTGTTGCTAGCAGGGTGTT |
| SNF2 Diagnostic R | CTTGATTATGTCCGCACGCC |
| SNF2 MX F | GAGGGATTAATGTTTGTCTACGTATAAACGAATAAGTACTTATATTGCTTTAGGAAGGTACCTTGACAGTCTTGACGTGC |
| SNF2 MX R | ATGAACATACCACAGCGTCAATTTAGCAACGAAGAGGTCAACCGCTGCTATTTAAGATGGCGCACTTAACTTCGCATCTG |
| SNF5 Diagnostic F | CAGGTGCTTGAAGGAGGGAG |
| SNF5 Diagnostic R | ACTGTTGTTGCTGTTGTCGC |
| SNF5 MX F | AAACACCAAAACAAAGCATCATCAAGGGAACATATAGTAAAGAACTACACAAAAGCAACACCTTGACAGTCTTGACGTGC |
| SNF5 MX R | TACAAATTCTTCCACGGTTATTTACATCTCCGGTATATTTTATATATGTGTATATATTTTCGCACTTAACTTCGCATCTG |
| SWR1 Diagnostic F | ACCCTTGTCTTTTGCAGCCT |
| SWR1 Diagnostic R | TGAACTGCGAACCTGGCTAG |
| SWR1 MX F | TGAAAATTTATGAATTCTAACTGCTCTTTGCATTTTCCAAGTTATTGCATTACAAGAATACCTTGACAGTCTTGACGTGC |
| SWR1 MX R | TCAATAATAATAACCGTTGGCAATAAACCTGATCATGTACTCGTCAACATGGGCAGTGCCCGCACTTAACTTCGCATCTG |
| TUP1 Diagnostic F | AAGCTCTCCCGTCAAAGCAA |
| TUP1 Diagnostic R | AGGAAAAGGAGGGGAAGGGA |
| TUP1 MX F | TTTTTTTGTCTTTTTTGATAAGCAGGGGAAGAAAGAAATCAGCTTTCCATCCAAACCAATCCTTGACAGTCTTGACGTGC |
| TUP1 MX R | ATGAATTGAAGAATAGTTTAGTTAGTTACATTTGTAAAGTGTTCCTTTTGTGTTCTGTTCCGCACTTAACTTCGCATCTG |
| Ctk1_ChrXI_check1_F | CCGCATTTACTGCACATCC |
| Ctk1_ChrXI_check1_R | GAAATTATACGCGGCAAGGA |
| Ctk1_ChrXI_check2_F | GCACCACACTAGCCTTCGAT |
| Ctk1_ChrXI_check2_R | CCAAAAGCAATCCAGGAAAA |
| Ctk1_ChrXI_check3_F | TTGTGGATTTCGGAGAAAGG |
| Ctk1_ChrXI_check3_R | AGGGAACACTCCGTCTGAAA |
| Ctk1_ChrXI_check_control1_F | GCACCCGAAAAATTAACTTGA |
| Ctk1_ChrXI_check_control1_R | TGGGATCAGAACAATCATTACAA |
| Gcn5_ChrIV_check1_F | GAACTGGCTCTCCCACAGTC |
| Gcn5_ChrIV_check1_R | GGACGAATCAACGGAGGGTT |
| Gcn5_ChrIV_check2.1_F | TCCGAATTGCCCGAAAATGC |
| Gcn5_ChrIV_check2.1_R | GACACCTTACGTTCTCGCCT |
| Gcn5_ChrIV_check2.2_F | AGGGTAATTCATCCTTCCACCA |
| Gcn5_ChrIV_check2.2_R | TCGAAAGCAAACTTGTGAAGGC |
| Gcn5_ChrIV_check2.3_F | TGCTTTTTCCCATCCCTGCA |
| Gcn5_ChrIV_check2.3_R | TCGCCGTTGTTCAAGACCTT |
| Gcn5_ChrIV_check3_F | CCCAAACTGATTGCCAAGCT |
| Gcn5_ChrIV_check3_R | TGGCACTAGCTGGTAGTAGC |
| Gcn5_ChrXII_check1_F | TCGGCCCAAACACCAGATTT |
| Gcn5_ChrXII_check1_R | CCCCTTTTCCTTCCTCGTCC |
| Gcn5_ChrIV_check_control_F | CATGGTCCAAGGTTGCAAGT |
| Gcn5_ChrIV_check_control_R | CCCGCCATCAATCATTGTGC |
| Gcn5_ChrXII_check_control_F | GTGCCATTCAAGTGCCCAATT |
| Gcn5_ChrXII_check_control_R | ACTTTCCCATCCGCATAAAGA |

**Supplementary Table 12. List of all primers used in this study.**

**Supplementary Notes**

**Supplementary Note 1. Conditional essentiality of *esa1Δ* segregants**. Esa1, the catalytic subunit of the NuA4 HAT complex, is essential in the BY background, with *esa1∆* spores only able to divide four or five times following germination before dying ^4^. However, we found that roughly 1% of the *esa1Δ* knockout segregants generated from the *ESA1* hemizygous diploids survive in our study. To examine for selection on specific genotypes in the *esa1*Δ segregants, allele frequency was examined across all SNP markers (**Supplementary Fig. 3a**). All *esa1∆* segregants carried the 3S genotype from positions 467,219 bp to 472,584 bp on Chromosome XIV, which is centered on *END3*, an EH-domain containing protein that functions in endocytosis and actin cytoskeleton formation. Two lines of evidence suggest that *END3* is the causal gene at this locus: *end3∆* and a temperature sensitive allele of *ESA1* were previously found to be synthetic lethal in the BY background ^5^, and *END3* is a major contributor to trait variation in the BYx3S cross ^2,3,6,7^. We note that this chromosome XIV locus was also identified as having a large additive effect in other knockout and wild type populations. We did not observe any other fixed regions in *esa1∆* segregants. This implies that conditional essentiality of *ESA1* may depend on the cumulative effect of the chromosome XIV region and many small effects variants.

**Supplementary Note 2. All major results are robust to different significance thresholds.** Significance thresholds can affect the results and interpretations of genetic mapping studies, especially those focused on genetic interactions. To address this possibility, we reiterated our work across a number of different False Discovery Rate (FDR) thresholds (**Methods**). Although choice of threshold impacted the number of genetic effects that were detected (**Supplementary Table 7**), we found that all of the major results remain the same regardless of threshold (**Supplementary Figs. 5** and **6**; **Supplementary Table 8** and **9**). This implies that our main conclusions are robust to threshold.

**Supplementary Note 3. All major results are also robust to bias in allele combinations.** In the paper, we show that mutation-independent effects tend to be genetically simpler, while mutation-responsive effects tend to be more genetically complex. This may be a technical artifact driven by allele frequency differences between the different knockout and wild type versions of the BYx3S cross, which has the potential to cause both false negatives and false positives. To examine this possibility, we excluded loci that show biased individual or multi-locus allele frequencies from our analyses. After exclusion, we found that mutation-responsive effects still involve more higher-order epistasis than mutation-independent effects (**Supplementary Table 10**). This difference was determined to be significant for both two-locus and three-locus mutation-responsive effects using chi-square tests (p-value = 6.731 x 10^-19^ and 5 x 10^-30^, respectively). Additionally, similar to analyses done with different significance thresholds, we find that our conclusions remain qualitatively the same when we include or exclude loci that exhibit allele frequency bias (**Supplementary Figs. 5** and **6**; **Supplementary Table 8** and **9**). Thus, this implies that our major findings are not the results of technical artifacts.

**Supplementary References**

1 Tong, A. H. & Boone, C. Synthetic genetic array analysis in *Saccharomyces cerevisiae*. *Methods Mol Biol* **313**, 171-192 (2006).

2 Taylor, M. B. & Ehrenreich, I. M. Genetic interactions involving five or more genes contribute to a complex trait in yeast. *PLoS Genet* **10**, e1004324 (2014).

3 Taylor, M. B. & Ehrenreich, I. M. Transcriptional derepression uncovers cryptic higher-order genetic interactions. *PLoS Genet* **11**, e1005606 (2015).

4 Smith, E. R. *et al.* *ESA1* is a histone acetyltransferase that is essential for growth in yeast. *Proc Natl Acad Sci U S A* **95**, 3561-3565 (1998).

5 Lin, Y. Y. *et al.* A comprehensive synthetic genetic interaction network governing yeast histone acetylation and deacetylation. *Genes Dev* **22**, 2062-2074 (2008).

6 Lee, J. T., Taylor, M. B., Shen, A. & Ehrenreich, I. M. Multi-locus genotypes underlying temperature sensitivity in a mutationally induced trait. *PLoS Genet* **12**, e1005929 (2016).

7 Taylor, M. B., Phan, J., Lee, J. T., McCadden, M. & Ehrenreich, I. M. Diverse genetic architectures lead to the same cryptic phenotype in a yeast cross. *Nat Commun* **7**, 11669 (2016).
