# Supplementary material for: The complex underpinnings of genetic background effects
Source: Nat Commun. 2018 Sep 17;9:3548. doi: 10.1038/s41467-018-06023-5 (PMC6141565; doi:10.1038/s41467-018-06023-5)
Supplement: Supplementary file 3 — Description of Additional Supplementary Files [file 41467_2018_6023_MOESM3_ESM.pdf]

Supplementary Data 1. Genotype table for the 1,411 segrega nts. Chromosome and Position columns correspond to the chromosome and position of genetic markers we used in this study, respectively. Column 3 through 1413 shows the haplotype of each segregant. 0 and 1 respectively denote a segregant carrying the BY and 3S alleles at a given marker.

Supplementary Data 2. Phenotype table. This table shows the growth of 3 biological replicates for each segregant across the 10 environments. Each replicate is labeled with a 1, 2, or 3. The mean growth of each segregant, which was used in linkage mapping, is also shown. If a segregant is missing a growth value due to technical issues, the mean was calculated using the two remaining biological replicates.

Supplementary Data 3. All identified genetic effects. This table shows all genetic effects that were identified in this study. Chromosome1 and Position1 columns indicate the chromosome and the peak position of the first locus in each genetic effect. Chromosome2, Position2, Chromosome3, and Position3 columns indicate the chromosome and positions of the second (in pairwise and three-way effects) and third (in three-way effects) loci in each higher-order genetic effect. The Environment column shows which environment the genetic effect was identified. Columns labeled as ctk1_pvalues, esa1_pvalues, gcn5_pvalues, htb1_pvalues, hos3_pvalues,

ino80_pvalues, or rpd3_pvalues correspond to the p-values found from pairwise contrasts between mutation and WT populations. Columns labeled as ctk1_contrast, esa1_contrast, gcn5_contrast, htb1_contrast, hos3_contrast, ino80_contrast, or rpd3_contrast indicate if the genetic effect interacts with the given knockout. 0 means no interaction (p-value > 0.05) and 1 means significant interaction was observed (p-value ≤ 0.05). If the genetic effect was identified as

mutation-independent, these columns are denoted as NA. If we were unable to conduct pairwise contrast due to fixed sites within a knockout population, then they were also denoted as NA. The Type column specifies if the genetic effect was found to be mutation-independent or mutation-responsive. The 99CI.start1 and the 99CI.end1 columns indicates the 99% confidence interval for the first locus in each genetic effect, while columns labeled with 99CI.start2, 99CI.end2, 99CI.start3, and 99CI.end3 shows the 99% confidence interval for the second and third locus in each genetic effect, respectively.

Supplementary Data 4. Phenotypic variance explained by all mutation-responsive two-locus interactions. This table shows all mutation-responsive two-locus genetic effects that were identified in this study. Chromosome1 and Position1 columns indicate the chromosome and the peak position of the first locus in each genetic effect, while Chromosome2 and Position2 columns indicate the chromosome and the peak position of the second locus. Environment column shows which environment the genetic effect was identified and Knockout columns shows which knockout the genetic effect interacts with. Column 7 through 9 represents the ratio of phenotypic variance explained by the individual loci (Ratio.locus1:knockout and Ratio.locus2:knockout) and their interaction (Ratio.locus1:locus2:knockout), respectively.

Supplementary Data 5. Phenotypic variance explained by all mutation-responsive three-locus interactions. This table shows all mutation-responsive three-locus genetic effects that were identified in this study. Columns Chromosome1, Chromosome2, and Chromosome3 specify the chromosome of the first, second, and third locus involved for each genetic effect. Similarly, columns Position1, Position2, and Position3 specify the peak position of the first, second, and third locus involved for each genetic effect. Environment column shows which environment the genetic effect was identified and Knockout columns shows which knockout the genetic effect interacts with. Column 9 through 11 shows the phenotypic variance explained by the cumulative effect of individual locus (Ratio.one-locus.interactions), the cumulative effect of all pairwise interactions of two loci (Ratio.two-locus.interactions), and the interaction of three loci (Ratio.three-locus.interaction), respectively.

Supplementary Data 6. The phenotypic variance explained by each mutation-responsive effect in wild type and relevant knockout segregants. This summary table consists of a list of all genetic effects detected in our study, as well as the phenotypic variance explained by these effects in wild type or knockout segregants. Chromosome1 and Position1 columns indicate the

chromosome and the peak position of the first locus in each genetic effect. If a genetic effect involved two- or three-way genetic interactions among loci, the chromosome and the peak position of the second locus are shown in the Chromosome2 and Position2 columns and the chromosome and the peak position of the third locus are shown in the Chromosome3 and Position3 columns. The Environment column shows which environment the genetic effect was identified. Columns labeled as ctk1, esa1, gcn5, htb1, hos3, ino80, or rpd3 indicate if the genetic effect interacts with the given knockout. 0 means no interaction and 1 means significant interaction was observed. Columns 15-22 (titled WT.VE through rpd3.VE) show the phenotypic variance explained by the genetic effect in each wild type or mutant population. We note that variance explained by a genetic effect was calculated in each background regardless of whether or not the effect was responsive to a particular knockout. However, in figures, we only show the results for genetic effects that respond to a given knockout. The Counts column shows the number of knockouts that each genetic effect interacted with.
